# Village sanitation and child health: Effects and external validity in a randomized field experiment in rural India

**DOI:** 10.1016/j.jhealeco.2016.03.003

**Published:** 2016-07

**Authors:** Jeffrey Hammer, Dean Spears

**Affiliations:** a Woodrow Wilson School, Princeton University, United States; b Economics and Planning Unit, Indian Statistical Institute – Delhi, India

**Keywords:** Sanitation, Health, Experiment, External validity, India

## Abstract

Over a billion people worldwide defecate in the open, with important consequences for early-life health and human capital accumulation in developing countries. We report a cluster randomized controlled trial of a village sanitation intervention conducted in rural Maharashtra, India designed to identify an effect of village sanitation on average child height, an outcome of increasing importance to economists. We find an effect of approximately 0.3 height-for-age standard deviations, which is consistent with observations and hypotheses in economic and health literatures. We further exploit details of the planning and implementation of the experiment to study treatment heterogeneity and external validity.

## Highlights

• Many people in developing countries defecate in the open without using a toilet.
• We study a field experiment of the effects of sanitation on child height in India.
• We find that the program caused children to grow taller, on average.

## 1 Introduction

Height has emerged as an important marker of human capital, attracting wide-ranging attention from economists (Steckel, 2009). This is because children who are able to grow to their height potentials are also able to develop towards their cognitive and other human capital potentials (Case and Paxson, 2008). One large threat to early-life growth in developing countries is poor sanitation, especially open defecation. More than one billion people worldwide defecate in the open without using a toilet or latrine. Open defecation is particularly widespread in India, and it has been suggested that this fact can help explain why children in India are among the shortest in the world (Spears, 2013). Especially because the public good nature of sanitation suggests an important economic policy role, it is therefore important to better understand any causal effect of exposure to poor sanitation on child health (Cutler and Miller, 2005).

This paper makes three contributions to the literature. The first and main contribution is to present results of a cluster randomized controlled experiment designed to estimate effects of rural sanitation on child height. In 2004, the government of Maharashtra in partnership with the World Bank conducted a randomized village-level sanitation promotion intervention. We document evidence indicating that the intervention caused a modest improvement in sanitation and an increase in child height. An effect of sanitation on child net-nutritional outcomes is consistent with evidence and theories from the medical and epidemiological literature, especially in India where high rural population density may worsen disease externalities.

A second contribution of the paper is to document evidence suggestive of externalities: we find apparent effects of neighbors’ latrine use even on households whose members continued to defecate in the open; a quantitative bounding exercise allows us to largely rule out that the latrine use of a child's neighbors did not, on average, matter for her height. Finally, the history of this experiment permits a third contribution to the economics of randomized field experiments, as they can applied in practice with the ability to critically analyze our experimental result and estimate its external validity where the experiment *did not* happen. Due to institutional features of the partnership between the World Bank and the government of Maharashtra – unrelated to the internal validity of our experiment – comparable data were simultaneously collected in other parts of Maharashtra where the government *considered* conducting an experiment, but where no attempt at an experiment was ultimately made. Although we, of course, cannot use these data to *know* what the effect of the experiment would have been in other parts of Maharashtra, variation within the district that we can study predicts that the effect might have been much smaller in the places which narrowly missed being selected for the experiment.

This paper proceeds in sections. Section 3 details the experimental method and empirical strategy, and Section 4 presents results. These estimates are important not only for assessing the impact of a part of a large government development program as implemented, but also for documenting that an improvement in sanitation can cause an improvement in child height. Next, Section 5 considers the external validity of this result, taking advantage of comparable data collection in two districts where the experiment did not occur. Section 6 concludes with a discussion of these results.

## 2 Sanitation, health, and early-life human capital

According to joint UNICEF and WHO (2012) estimates for 2010, 15% of the world population and 19% of people in developing countries defecate in the open without using any toilet or latrine. Of these 1.1 billion people, nearly 60% live in India, which means they make up more than half of the population of India. People in India are much more likely to defecate in the open than even people in much poorer sub-Saharan African countries, on average, and open defecation in India has declined little despite rapid economic growth (Coffey et al., 2014).

On average, Indian children are exceptionally short; because height is an important indicator of human capital, the puzzle of widespread stunting in India has attracted the recent attention of many economists (*e.g.* Deaton, 2007, Tarozzi, 2008, Jayachandran and Pande, 2013). Although stunting is commonly referred to as an indicator of “malnutrition,” evidence is accumulating for an important role of the disease environment in shaping nutritional outcomes (Smith et al., 2013). For example, the economic history literature has shown a large association between average population-level heights and the disease environment, as reflected in mortality rates (Bozzoli et al., 2009). Hatton (2013), studying the historical increase in European height, concludes that “the most important proximate source of increasing height was the improving disease environment as reflected by the fall in infant mortality.”

Medical and epidemiological literatures describe at least four pathways by which disease from environmental fecal pathogens could reduce early-life growth: loss of nutrients due to diarrhea, energy expenditure fighting disease, worm and parasite infections, and malabsorption due to inflammatory responses of the intestine to repeated infection (Checkley et al., 2008). Most recently documented in detail in the medical literature, but perhaps very quantitatively important, is the possibility of chronic but subclinical “environmental enteric dysfunction,” which would reduce nutrient absorption and could cause stunting without causing diarrhea (Humphrey, 2009).^1^

Non-experimental econometric evidence is consistent with an important effect of poor sanitation on early-life health and child human capital (*e.g.* Galiani et al., 2005). For example, Cutler and Miller (2005) find a large effect of water filtration and chlorination on mortality in major U.S. cities in the early 20th century. Bleakley (2007) documents that eradicating hookworm infection – one of the several mechanisms by which poor sanitation impacts health – improved learning and increased incomes in the American South. Spears (2013) has recently observed that heterogeneity across developing countries in open defecation rates can explain a large fraction of the variation in average child height. Geruso and Spears (2015) exploit a difference in demand for latrine use between Hindus and Muslims within India to document an effect of local open defecation on infant mortality.

It is therefore of high importance to both economists and policy-makers to make well-identified estimates of the causal effect of sanitation on child height. This study presents what was, to our knowledge, the first randomized controlled trial of the effect of village sanitation on child height. We study data from a village-level sanitation program, implemented in 2004 in the context of Maharashtra's phased roll-out of the Indian government's national Total Sanitation Campaign (TSC). A senior official of the Maharashtra government chose to collaborate with the World Bank to exploit the initial phase-in of the TSC to conduct an impact evaluation. Alok (2010), in his memoirs as an administrative officer responsible for the TSC, describes Maharashtra as an early and rapid adopter of the TSC. The period that we study is therefore very early in the implementation of the TSC, when there would have been a national sanitation *policy*, but this would not yet have been widely effectively implemented in *programs*. The period we study would also have been effectively before the Clean Village Prize, a part of the TSC which is exploited in Spears’ (2012a) identification strategy. Because this was the initial implementation of TSC programs in these districts of Maharashtra, despite the existence of a written national sanitation policy, there was little risk of “contamination” of the control group during the period studied.

## 3 Empirical strategy

In 2004, the government of Maharashtra, in collaboration with the World Bank Water and Sanitation Program, conducted a sanitation promotion intervention, randomly allocated at the village level. We use the experiment to learn about the effect of rural sanitation on early-life human capital accumulation. The timeline of this experiment contained four events: the experimental intervention in early 2004 and three survey rounds.

• February 2004: baseline survey data collection,
• Shortly thereafter: village-level sanitation “triggering” intervention,
• August 2004: midline survey data collection,
• August 2005: endline survey data collection,

Therefore, about 18 months elapsed between the experimental intervention and the final observations of outcomes.

### 3.1 The program: latrine construction and village sanitation promotion

The experimental program studied here was conducted in the context of the initial introduction of India's Total Sanitation Campaign (TSC) by the Maharashtra state government. The TSC was a large government effort throughout rural India, partially funded by the central government, but implemented by state governments.^2^ The program studied in this paper is a randomized initial implementation of the TSC in one district; that is, the state and district governments randomly selected villages to receive the TSC *first* in one district. Because the TSC was only just beginning in Maharashtra at this time, villages randomly assigned to the control group were receiving no government sanitation program at all during the experiment.

The experimental program studied here had two components: (1) subsidized construction of brick household pit latrines by local governments, and (2) village-level sanitation motivation by a representative of the district government. Because these were provided as a combined programmatic package, we are unable to distinguish the effects of latrine construction and promotion. Inspired by the procedures of the Community-Led Total Sanitation (CLTS) movement, the program sent a sanitation promoter to visit the village and convene a series of meetings where information, persuasion, demonstration, and social forces were employed in an attempt to “trigger” a community-wide switch to latrine use. In general, promotion meetings attempted to use emotions such as disgust and shame to promote latrine use, including socially conservative values. For example, high caste villagers were encouraged to build latrines for and promote use by low caste villagers by a demonstration that flies move from feces to food (with the implication that everybody was eating low caste people's feces).^3^ This sanitation promotion was not intended to promote health in general or improve beliefs about health production more generally; results in Supplementary Appendix A1 verify that the treatment had no effect on knowledge about causes or treatment of diarrhea.

In addition to promotion of the use of latrines, the program organized and funded the construction of household pit latrines. In general, latrines were constructed throughout a village at one time, were made of brick, and had a single pit below a cement slab on which the user would squat. A small minority of households already owned latrines, although even in households which owned latrines, it would have been common before the program for only some household members to use them. An average TSC latrine in Maharashtra costs about $80 PPP (World Bank, 2010a). Among those who took up the program in treated villages, a standard TSC latrine was provided fully subsidized and free of charge, but some households spent additional money to modify or customize their TSC latrines.

#### 3.1.1 The TSC and sanitation promotion in the literature

Other studies in the literature about the TSC in rural India indicate that the TSC was, on average, able to achieve latrine construction and, in some cases, change behavior, although to a degree far short of elimination of open defecation. These studies can be thought of as independent evidence that TSC type activities did, in some contexts, have a “first-stage” effect on sanitation, even if a limited one.

For example, in the context of India's TSC, Pattanayak et al. (2009) find in a randomized, controlled trial in two blocks in a district of Orissa that in villages receiving a social “shaming” treatment (similar to the community meeting methods used in Maharashtra), latrine ownership increased from 6% to 32%; over the same time period, there was no increase in ownership in control villages. Barnard et al. (2013) conducted a cross-section survey of villages in which the TSC had been conducted in Orissa and similarly found moderate improvements in sanitation. Their conclusion is worth quoting in full: “A large-scale campaign to implement sanitation has achieved substantial gains in latrine coverage in this population. Nevertheless, gaps in coverage and widespread continuation of open defecation will result in continued exposure to human excreta, reducing the potential for health gains” (p. 1).

In a recently published medical study, Patil et al. (2014) report effects of another randomized implementation of India's TSC, in Madhya Pradesh in 2009; we recommend reading our study and theirs together, as well as Gertler et al.'s (2015) recent econometric analysis of these data. Patil et al. explain that, as is common in rural India, open defecation proved difficult to change in the first-stage: “the intervention led to modest increases in the availability of individual household latrines and even more modest reductions in open defecation.” Therefore, they do not detect any effects on child height.^4^

### 3.2 An experiment in one district of Maharashtra

Districts are the administrative unit of the Indian government that make up states. When the government of Maharashtra and the World Bank initially decided to conduct this experiment, they identified three districts: Ahmednagar, Nanded, and Nandurbar.

Table 1 compares the three districts with average properties of rural Maharashtra and all of rural India, using census and related data sources that are independent of this experiment. In general, Nandurbar appears poorest and has a larger Scheduled Tribe population, Nanded is in the middle, and Ahmednagar enjoys the best human development.^5^

Although high-level policy-makers in the government of Maharashtra originally planned to implement an experiment in all three districts, in fact, the government ultimately only attempted to implement the experiment in one district, Ahmednagar.^6^ In this district, the program was indeed implemented in 30 villages randomly selected out of 60 eligible for the treatment or control groups.^7^ No significant sanitation program, and certainly no part of the experimental program studied here, was implemented in Nanded or Nandurbar during this time. However, by the time it was settled that the government of Maharashtra would only attempt to implement the program in one district, the World Bank had already contracted with an independent survey organization to collect data in all three districts. Therefore, the data collection continued in all three districts. This change of initial plans, and seemingly unnecessary data collection, presents an unusual econometric opportunity to consider the external validity of experimental estimates and the implications of the often undocumented mechanisms by which experimental contexts are determined.

### 3.3 Dependent variable: child height-for-age

Physical height is a persistent summary measure of early-life health; early-life height predicts adult height (Schmidt et al., 1995), as well as human capital and economic productivity (Case and Paxson, 2008, Spears, 2012b, Vogl, 2014). Height of children under 5 is, therefore, the central dependent variable in our analysis. Indeed, a document by the original World Bank research team specified child height as the *only* health outcome where the original experimenters expected to find effects, in the sense of an informal pre-analysis plan.

In particular, surveyors were directed to measure the height of all children under five in a randomly selected 75% of households in each village surveyed.^8^ This age group is the focus of WHO growth reference charts; it is the age group measured by the Demographic and Health Surveys; and it is a commonly selected population in height studies. As Section 4.1 discusses, this means that children age out of and are born into our sample; although the sample was constructed to be a village-level panel of the average height of children under 5, in a robustness check we additionally show within-child results on the growth trajectory of children who were young enough at baseline to be measured in all three survey rounds.

In our main results, we transform height into *z*-scores using the 2006 WHO reference population. However, our results are robust to using log of height in centimeters as the dependent variable instead; certain specifications are also robust for using dichotomized stunting^9^ as the dependent variable, but the use of this measure is well understood in the literature to sacrifice statistical power. In our conversion of raw height data into height-for-age *z*-scores, we use the Stata user-written command zscore06 by Jef Leroy; use of this conversion software is standard in the literature and it is frequently cited.^10^ We find a highly dispersed sample of height-for-age *z*-scores, relative to the WHO healthy reference population, with many very short children. In order to better understand this feature of our data, we compare the dispersion of our data to that in the Demographic and Health Survey (DHS) for India and the India Human Development Survey (IHDS), in an analysis presented in Supplementary Appendix B. By several measures, our data are less dispersed with fewer extreme values than the rural IHDS. Our data are more dispersed than the rural DHS, but when zscore06 is equivalently applied to raw DHS height data, the resulting *z*-scores are more comparable to ours than are the *z*-scores included in DHS data, with more apparently extremely short children.

In response to this issue, we truncate our sample to include only children with height-for-age between −8 and 4, approximately ±6 standard deviations around the average. No cut-points that we are aware of were specified in any pre-analysis plan by the original World Bank researchers. Results in Supplementary Appendix A compare estimates using these cut-points with estimates from 36 other combinations of upper and lower cut-points, and find that our conclusions are qualitatively robust, although not precisely constant across combinations of cut-points. Supplementary Appendix B, which focuses on the dispersion in our height data, further considers these truncation points, in comparison with other data sets and other possible modeling decisions. We verify that our result is not driven by extreme or influential height outliers.

Ultimately, we cannot conclusively verify the quality of our height data: as in many studies, we have no record of the surveyor–respondent interaction beyond what has been entered into our data from what was written on the survey form. The quality of these data is an important input into the trustworthiness of this study, so we encourage interested readers to consult these appendices.

### 3.4 Regression specification

Our preferred specification is a difference-in-differences at the individual child level, using only data from Ahmednagar district:

(1) $$z_{\mathrm{ivt}}=\beta_{1}{\mathrm{treatment}}_{v}+\beta_{2}{\mathrm{treatment}}_{v}\times {\mathrm{midline}}_{t}+\beta_{3}{\mathrm{treatment}}_{v}\times {\mathrm{endline}}_{t}+A_{\mathrm{ivt}}\Gamma +\alpha_{v}+\gamma_{t}+{ɛ}_{\mathrm{ivt}},$$

where *i* indexes individual children, v indexes villages, and *t* indexes the three survey rounds: baseline, midline, and endline. The dependent variable *z* is the child's height-for-age *z*-score, ${\mathrm{treatment}}_{v}$ is an indicator for living in a village assigned to the treatment group (it is only indexed by village), and *midline*_*t*_ and *endline*_*t*_ indicators for survey round. *β*_2_ and *β*_3_ are experimental effects. Survey round fixed effects *γ*_*t*_ will always be included, and to this a set of 120 age-in-months-times-sex indicators^11^ *A*_*ivt*_ and village fixed effects $\alpha_{v}$ will be added in stages to demonstrate that they do not change the result. We replicate the result using a similar specification

(2) $$z_{\mathrm{ivt}}=\beta_{1}{\mathrm{treatment}}_{v}+\beta_{2}{\mathrm{treatment}}_{v}\times {\mathrm{after}}_{t}+A_{\mathrm{ivt}}\Gamma +\alpha_{v}+\gamma_{t}+{ɛ}_{\mathrm{ivt}},$$

where the *midline*_*t*_ and *endline*_*t*_ indicators have been collapsed into the single variable *after*_*t*_, which is 1 for observations in the midline or endline survey round and 0 for observations in the baseline survey round. As a further robustness check, exploiting all of our data and the fact that no experimental intervention occurred in Nanded or Nandurbar, we use a triple difference, comparing the effect of random assignment to the treatment group in Ahmednagar to the effect of assignment in the unexposed districts. In this case, the estimate of the treatment effect is the triple interaction on Ahmednagar × treatment assignment × after, labeled *β*_6_ here:

(3) $$z_{\mathrm{ivt}}=\beta_{1}{\mathrm{treatment}}_{v}+\beta_{2}{\mathrm{treatment}}_{v}\times {\mathrm{after}}_{t}+\beta_{3}{\mathrm{Ahmednagar}}_{v}+\beta_{4}{\mathrm{treatment}}_{v}\times {\mathrm{Ahmednagar}}_{v}+\beta_{5}{\mathrm{after}}_{t}\times {\mathrm{Ahmednagar}}_{v}+\beta_{6}{\mathrm{treatment}}_{v}\times {\mathrm{after}}_{t}\times {\mathrm{Ahmednagar}}_{v}+A_{\mathrm{ivt}}\Gamma +\alpha_{v}+\gamma_{t}+\delta_{v}+{ɛ}_{\mathrm{ivt}},$$

where $\delta_{v}$ are added as district fixed effects in specifications where village fixed effects $\alpha_{v}$ are not used.

Because the experimental treatment was assigned at the village level, in all regression estimates we calculate standard errors clustered by village. In Ahmednagar, there are 60 surveyed villages, which exceeds Cameron et al.'s (2008) threshold of 50 clusters for reliable standard errors.

### 3.5 The Clean Village Prize: a measure of implementation

As a subsequent part of its Total Sanitation Campaign, the central Indian government awarded villages a Nirmal Gram Puraskar (Hindi for Clean Village Prize) in recognition of becoming open defecation free. Villages certified by central government auditors to be open defecation free receive a trophy and a cash prize, presented to the village chairman at a prestigious ceremony in the state or national capital (World Bank, 2010b, Lamba and Spears, 2013). Although only about 4% of all Indian villages have won the prize, this number is much larger in Maharashtra, where over 9000 prizes have been won, more than any other state and, indeed, about one-third of the total number of prizes awarded.

The Clean Village Prize was implemented in Maharashtra *after* the experiment we study. We therefore treat receipt of the Clean Village Prize as an additional *measure* of village sanitation coverage that is independent of data collection by the survey company contracted by the World Bank. We obtained administrative records from the Indian central government on which villages in Ahmednagar had ever won the clean village prize by mid-2012. Our data request to the central government made no reference to this experiment. Prizes were first awarded in 2006 to any of the villages we study; therefore, village governments would be unlikely to have heard of the prize at the time of our experiment, and the prize almost certainly had no influence on the experiment. Through the summer of 2012, 12 of the 60 villages studied in Ahmednagar had won the prize. To verify that an experimental implementation occurred, we will investigate whether villages assigned to the treatment group were more likely to go on to win this sanitation prize.

## 4 Results

This section presents results. First, the experiment balanced observed baseline properties. Second, the experiment improved sanitation coverage, but did not eliminate open defecation. Third, in an independent measure of this effect on sanitation; villages assigned to the treatment group were more likely to subsequently win a central government prize for being open defecation free. Fourth, we show an effect on survey-reported diarrhea morbidity among the children whose height we study. Finally, in our main result, we show a statistically robust effect on children's height.

### 4.1 Sample and balance of observed baseline properties

Did the random assignment of villages to treatment and control groups achieve balance on observed baseline characteristics? Table 2 shows that the answer is yes, both for the district Ahmednagar where the program was implemented, and for the other two districts. Across a range of variables, in no case is there a statistically significant difference between the assigned treatment and control villages in variables observed in February 2004, before the program. Households in the treatment and control groups are similar in the first and second principal components of a vector of assets asked about in the baseline survey. The summary statistics in the table reflect the poverty and poor health in the studied districts. As an illustration of their poverty, we note that only about three-fourths of households owned a clock or watch.

Table 2 describes how the sample is distributed across the three survey rounds, in Ahmednagar. Of the 3432 observations, 771 of them were children who were observed and able to be matched in the data across all three survey rounds. The remaining children appeared in either one or two survey rounds. Just as in the widely used Demographic and Health Surveys, data collection for this experiment only measured the height of children under 5. Therefore, child age is correlated with appearances across survey rounds, as shown in panel B of Table 3. Children who appeared in all three survey rounds started young in the first survey round and grew older; children who only appeared in one survey round are old in round 1 (they age out of being under 5) but young in round 3 (they are relatively newly born, into the potentially healthy disease environment). Regression controls for 120 indicators of age-in-months by sex ensure that our result is not due to a mechanical association between child age and average height-for-age.

One potential concern would be differential attrition in the treatment and control groups. However, the balance Table 2 verifies that this did not occur. Treatment group children did not appear in any more survey rounds, on average, than control group children; indeed, treatment group children were almost precisely as likely in Ahmednagar to be measured in all three rounds as were control group children. This study was designed as a panel of villages, not a panel of children: the original World Bank research team intended to learn about the effect of the disease environment on average child height; because emerging evidence suggests that sanitation can begin to influence child height *in utero* (Prendergast et al., 2014), and because much of the variation in child height is already determined by the time she is 5 years old, it is appropriate that, following the DHS procedure of measuring children under 5, older children were replaced with younger children to measure this population-level average outcome. That said, as a robustness check Table 6 will focus on a restricted sample of children measured in multiple rounds or all three rounds, to show that results using child fixed effects to study changes in the growth trajectories of children are quantitatively similar.

### 4.2 First stage: effect on sanitation

Did the program indeed have an effect on sanitation? As panel A of Table 4 shows, respondents in treatment group villages in Ahmednagar, in the midline survey after implementation, are more likely to report a visit by a sanitation promoter and are more likely to have a household latrine. This difference is marginally statistically significant.^12^ As expected, no such differences are seen in the two districts where data were collected, but no experiment was attempted.

Perhaps more importantly, villages in the treatment group built more latrines. In the final survey round in August of 2005, treatment village household latrine coverage in Ahmednagar had increased by 8.2 percentage points more than for control households. The Ahmednagar difference has a two-sided *p*-value of 0.073 and 0.072 in household-level and village-level regressions, respectively.

**Fig. 1:**
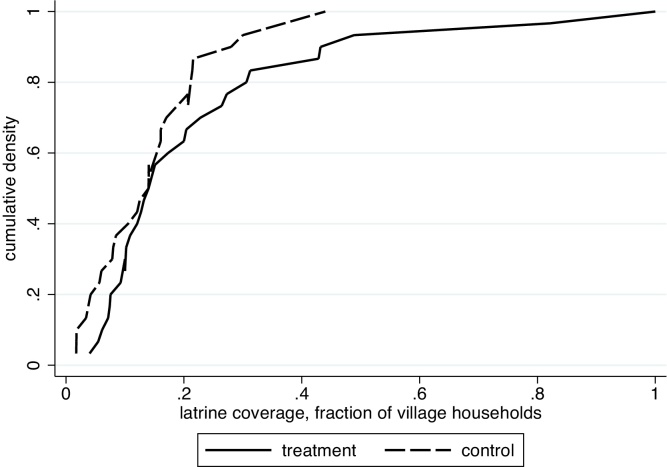
Distribution of village-level sanitation in Ahmednagar district, endline survey.

The distributions of village sanitation coverage in the treatment and control groups are different throughout, that is, they do not only differ among, for example, villages with high or low sanitation coverage. Fig. 1 plots the CDFs of village latrine coverage for the treatment and control groups in Ahmednagar in the endline data. The figure highlights that only a few treatment group villages achieved more than 50% coverage. Thus, even villages that fell far short of eliminating open defecation had some improvement in sanitation.

For both intermediate outcomes (that is, recollection of promotion visits and household latrines), there was no corresponding effect in Nanded and Nandurbar districts. This is expected because (although “treatment” and “control” statuses were unnecessarily assigned) the government did not attempt to implement any part of the program there during the period studied; we include this verification as a placebo test.

#### 4.2.1 How large is the first-stage effect on sanitation?

How large is the apparent effect on sanitation? It appears modest; an 8.2 percentage point increase left many people defecating in the open. However, part of the community sanitation promotion effort was to persuade some households to use latrines they already owned, which could increase the effect on latrine *use* above the effect on latrine ownership.^13^ The medical and epidemiological literature reports detectable effects on intermediate health outcomes of similarly moderate changes in sanitation.

Indeed, one important implication of this modest improvement is that it is very difficult to improve rural sanitation, perhaps especially in India, even as part of a special government–World Bank experimental partnership. This effect size is similar to other causally well-identified impacts on rural sanitation of other programs studied in the literature as cataloged in Panel B of Table 4; each study reflects an important intervention by the Indian government, the World Bank, or both. Of six effect sizes, three are larger and three are smaller than this experiment's effect on latrine ownership, and none finds an effect on latrine ownership larger than 20 percentage points, or on latrine use much larger than 10 percentage points.

### 4.3 Verifying an effect on sanitation with prize data

From the central government, we received data on village sanitation prize winners in July 2012, indicating which villages in Ahmednagar had ever won the prize by that time. In the treatment group, 9 of 30 villages have won the clean village prize; in the control group, 3 of 30 villages have won the prize. This 20 percentage point difference is statistically significant.^14^ Because these prizes were awarded several years after our experiment ended, because they involve several investigations by various agents, and because during the time period studied prizes were ultimately approved by the *central* government in Delhi rather than the state government, we consider it to be very unlikely that the prize outcomes were manipulated to create the appearance of an effect of this experiment (the data from which was essentially abandoned for several years). Therefore, we interpret this finding that treatment group villages were more likely to go on to win the prize than control group villages, as additional confirmation that the experiment happened and caused an improvement in sanitation.

### 4.4 Survey-reported diarrhea morbidity

Although the main outcome intended by the original research team was child height-for-age, survey questions asked mothers to report whether their children had experienced diarrhea or cough in the past two weeks.^15^ Although it is now increasingly understood that mechanisms such as environmental enteric dysfunction could influence child height without manifesting as diarrhea (Humphrey, 2009), we analyze this survey-reported morbidity data as a mechanism and plausibility check on our main result.

**Fig. 2:**
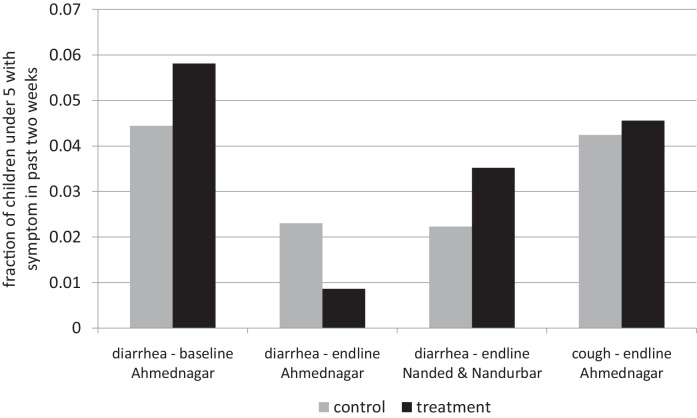
Survey-reported morbidity by assigned treatment status.

Fig. 2 presents effects of the experiment on reported diarrhea morbidity. Within Ahmednagar, there is no difference between treatment and control groups at baseline (*t* = 1.07; *p* = 0.29); but at endline, reported diarrhea was statistically significantly reduced in the treatment group relative to the control group (*t* =−2.12; *p* = 0.04). Thus, the difference-in-differences estimate of the impact of the program on survey-reported diarrhea is a reduction of 2.8 percentage points (*s* . *e* . =0.013; *p* = 0.029). As a plausible indicator of mechanism specificity, following Galiani et al. (2005), there is no impact in reported cough (*t* = 0.24; *p* = 0.82), nor of planned treatment and control assignment in Nanded and Nandurbar.

### 4.5 An effect on child height

Table 5 presents regression evidence from Ahmednagar that the experimental program increased child height, on average. The table reports results from 12 specifications in order to demonstrate the robustness of the finding. Results are collected into four panels, corresponding with regression Eqs. (1)–(4), respectively:

• *Panel A:* Double difference (Ahmednagar only, treatment × time), midline and endline separated, that is, treatment and control villages were compared only using Ahmednagar data, comparing the differences over time between the two groups.
• *Panel B:* Double difference (Ahmednagar only, treatment × time), midline and endline collapsed into “after.”
• *Panel C:* Triple difference (Nanded and Nandurbar included, treatment × time × Ahmednagar), midline and endline separated, that is, including all villages in the sample (not just in Ahmednagar), with the difference between treatment and control time trends also being compared across districts.
• *Panel D:* Triple difference (Nanded and Nandurbar included, treatment × time × Ahmednagar), midline and endline collapsed into “after.”

Within each panel, three specifications are included:

• *Column 1:* The basic double or triple interaction, and nothing else.
• *Column 2:* To column 1, we add 120 dummies for age in months 1–60, separately for boys and girls. This accounts for the unfolding of stunting over time, for any mean differences between our population and the WHO reference population, and for any differences in age structure across experimental groups. Adding these controls slightly increases the experimental point estimate in two cases and decreases it in two cases, but in no case makes an important difference.
• *Column 3:* To column 2, we add village fixed effects (constant across the three survey rounds). Because the treatment was randomly assigned to villages, we would not expect these to have an effect, and they do not, other than to slightly reduce standard errors.

In all cases an effect of the program is seen, typically in the range of 0.3–0.4 height-for-age standard deviations, or about 1.3 cm in a four-year-old. McKenzie (2012) recommends longer time series in experimental studies than simple before-and-after. Although we only have two post-intervention survey rounds, it is notably consistent with our interpretation of the results as representing an effect of the program that the point estimate for the endline is greater than the point estimate for the midline in every case, perhaps as the effects of reduced enteric infection have had an opportunity to accumulate. So, in panel A, the effect ranges from 0.236 to 0.278 at midline, and from 0.379 to 0.448 at the end. Without making the distinction of endline to midline (that is, ignoring the length of exposure to the program in panel B), the effect is unsurprisingly in the middle: 0.324–0.357. A further alternative specification is to omit any use of *z*-scores by using height in centimeters as the dependent variable, in logs to account for different effect sizes at different ages. The effect of the program in the endline period is to increase height by 1.8% (*t* = 2.20) in the double difference (comparable in functional form to column 2 of panel A).^16^

A final test responds directly to the concern that the overall result could be driven by one village with a large potential treatment effect or other special properties. We replicate the estimation of the “after” treatment effect in Ahmednagar 60 times, omitting each village in turn. The point estimate ranges from a minimum of 0.28 to a maximum of 0.37 and the *t*-statistic ranges from 1.94 to 2.66, with a mean of 2.20. Thus our result does not merely reflect any one outlier village.

#### 4.5.1 Improvement in height, but not up to healthy norms

How large is the estimated effect on children's height? One way to understand the effect is to compare it with Spears’ (2012a) estimates of the effect of the government's Total Sanitation Campaign throughout India. Averaging over incomplete and heterogeneous implementation throughout rural India, Spears finds that, on average, the program increased height-for-age *z*-scores by about 0.2 standard deviations. Our experimental estimates are about 1.5–2 times as large.

**Fig. 3:**
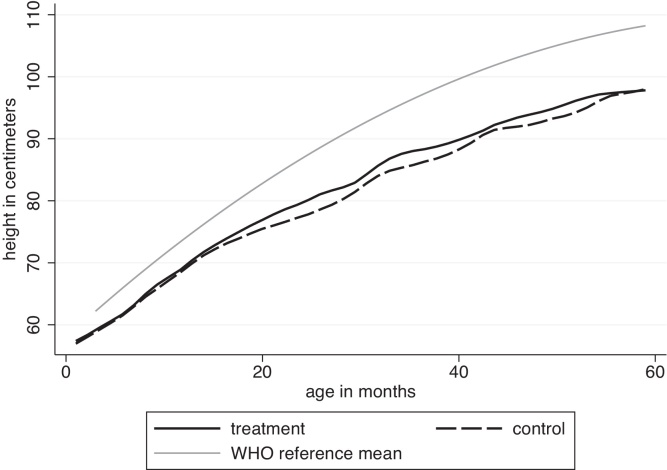
Height of children in Ahmednagar district by age, endline survey.

Another way to understand the effect size is to compare it with the gap between the average Indian child and the WHO reference population mean. On average, Indian children older than 24 months are about two standard deviations below the WHO reference mean, and the children in our study are even shorter. Fig. 3 plots the average endline heights at each age in the treatment and control groups in Ahmednagar (as kernel-weighted local polynomial regressions), alongside the mean height of the WHO reference population.^17^ The waviness in the graph is due to age heaping of children at round ages. The figure shows that treatment group children are taller than control group children, although not by nearly enough to reach the WHO reference mean.^18^

#### 4.5.2 Within-child differences in growth trajectories

As described in Section 4.1, because the experiment was intended to learn the effect of the village-level disease environment on village-level average height, and because this is chiefly malleable in early life, the height sample followed the DHS in measuring children under 5 at the time of the survey, rather than following a panel.^19^ However, 22% of the observations belong to children who were young enough in the initial survey to have their early-life growth traced through all three survey rounds. As a robustness check, this section concentrates on those children, to study the effect of open defecation on within-child differences in growth trajectories.

**Fig. 4:**
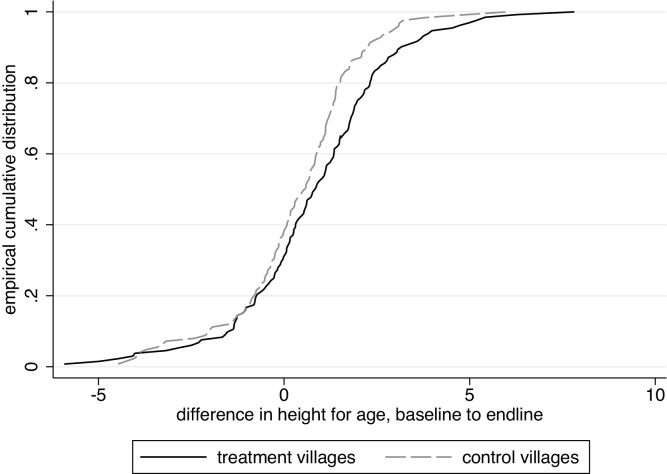
Within-child baseline-endline difference in height-for age, Ahmednagar district.

Fig. 4 plots the empirical cumulative distributions of the change between the endline and baseline surveys for these initially young children. The distribution for the treatment group is visibly to the right of the distribution for the control group. A Kolmogorov–Smirnov test rejects that these are the same distribution (*p* = 0.024).

Table 6 reports regression results for this sub-sample of a child-level panel. Column 1, for comparison, repeats column 1 of Table 5, the main result on the full sample. Column 2 restricts this sample to children who appear in all three survey rounds: although the estimates lose some precision due to the smaller sample, they are essentially unchanged. Column 3 verifies that adding child fixed effects does not change this estimate in the balanced panel.^20^ Columns 4 and 5 consider the sample of children who appeared in the data 2 or 3 times; again the results are essentially similar and not statistically significantly different from those for the full sample.

Just as economic data are sometimes dichotomized into an indicator for poverty, low height-for-age is sometimes dichotomized as *stunting*, an indicator of clinical significance that height-for-age is below -2 standard deviations. Spears et al. (2013) use Monte Carlo simulations to show that using dichotomized stunting as a dependent variable instead of continuous height-for-age reduces statistical power. However, we replicate our results using stunting as a dependent variable as a robustness check. In the full sample, the effect in Ahmednagar is a 7.0 percentage point decline in stunting (*p* = 0.16); in the sample of children observed for more than one survey round, it is a 14.6 percentage point reduction (*p* = 0.01); these two effect estimates (7.0 and 14.6) are not statistically significantly different from one another. For more details, see Supplementary Appendix A3.

#### 4.5.3 Negative externalities: effects in households without latrines

Existing observational evidence suggests negative externalities, effects of one household's open defecation on another's children (Spears, 2012a, Spears, 2013). However, these studies were not based on a randomized intervention study. In our study, children living in villages with more sanitation coverage grew taller than children living in other villages, on average (see Supplementary Appendix A2). An effect of the program we study on the heights of children whose households did not use latrines, even at the endline after the program, could suggest spillovers of sanitation onto other local households – however, as we will discuss, this comparison cannot rely on randomization for identification.

Indeed, even after the program most children lived in households without latrines. Restricting the sample to this subset^21^ (74.6% of the Ahmednagar sample) and estimating the simple difference-in-differences in panel B of Table 5 finds that the program caused even children in this group to be 0.42 standard deviations taller (standard error = 0.19, *n* = 2562). When the full sample is used with a fully-interacted triple difference, the effect of the program on households with a latrine at endline is no different than the effect on households without a latrine at endline: the estimate of the triple difference (treatment × after × own household latrine at endline) is 0.001 with a standard error of 0.20 and a *t*-statistic of 0.01. Therefore, this community-level experiment suggests spillover effects of open defecation.

To emphasize, this comparison does not benefit from the village-level random assignment. Consider three types of households: those who would choose to have toilets with or without the treatment, those who would have toilets only with the treatment, and those who would not have toilets with or without the treatment. The set of households who do not have a toilet in the treatment group includes only households that would not have a toilet with or without the treatment; the set of households without a toilet in the control group includes these households *and* those who would have switched, if exposed to the treatment. If this group is very different, self-selection could bias this result.

However, we can bound this bias, because we know from our first-stage results that the set of households who would switch into latrine ownership under the program is relatively small.^22^ For a simple computation, consider the case in which approximately 10% of households are switchers, while 10% would use latrines with or without the treatment and 80% would not. For the 0.42 standard deviation effect on non-owners to be due entirely to the composition effect of the small number of untreated would-be switchers (that is, the 10%) — and therefore for there to have been no effect greater than 0 on the height of non-owners — never-owners’ children would have to have experienced a change in height-for-age across the survey rounds that was over 4 *z*-score points greater (that is, more positive) than the change among children in households that would have switched if they had been assigned to the treatment. This is an implausibly large difference.^23^ Therefore, we believe that this quantitative bounding is reason to believe that the effect on non-owners is greater than 0: that there are positive externalities of village sanitation.

#### 4.5.4 Differences throughout the height distribution

Where the final differences between the treatment and control groups concentrated on taller or shorter children? Randomization only ensures an unbiased estimate of the *average* treatment effect, not of the full distribution of outcomes or treatment effects; but recognizing this, it still could be informative to compare the height distributions in the treatment and control groups.

**Fig. 5:**
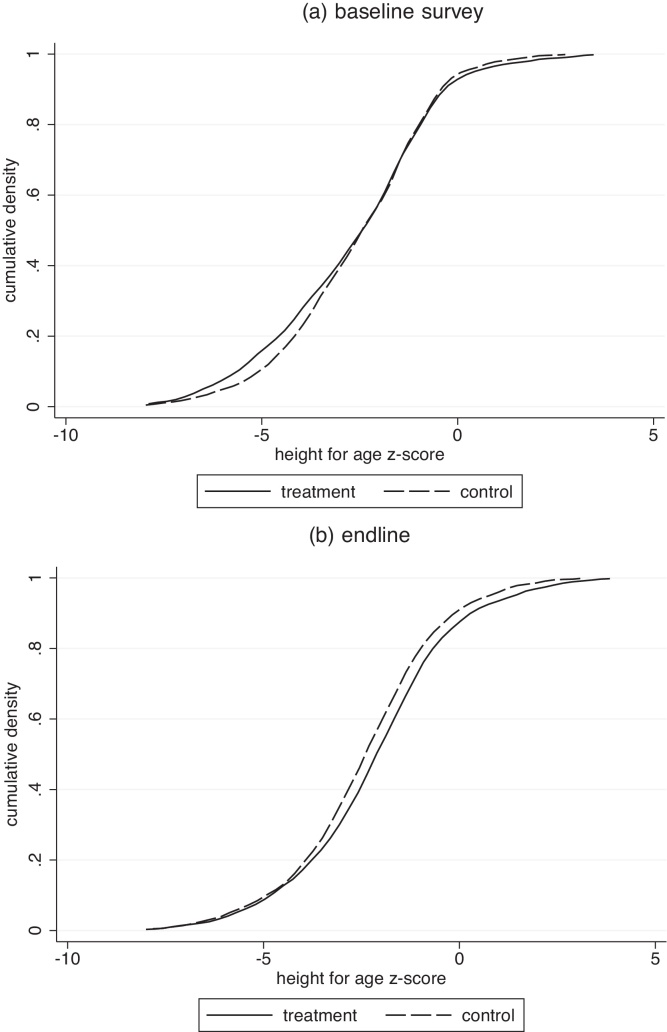
Distribution of height of children in Ahmednagar district.

Panel A of Fig. 5 plots height CDFs in the treatment and control groups in Ahmednagar in the baseline data, from before the program. The lines are very close to each other, as we would expect, with the slight separation at the bottom suggesting that the shorter children in the control group were not as short as the shorter children in the treatment group before the program.

Panel B presents the same CDFs from the endline data, after the program. Almost throughout the range, the treatment group distribution has moved to the right of the control group distribution. This suggests that improved sanitation moved both relatively tall and short parts of the height distribution. If so, this may be consistent with open defecation being a public bad with consequences for many people. A Kolmogorov–Smirnov test for equality of distribution rejects that the treatment and control distributions of height are the same after the program (*p* = 0.03), but does not use the data from before (*p* = 0.23).

## 5 External validity?

Economists have long recognized that program effects differ across people and places, and have tested for and modeled effect heterogeneity (Heckman, 2001). A well-conducted field experiment can provide an estimate of the average causal effect of a program in the population eligible for randomization. However, the wide use of experimental methods among economists has recently sharpened this concern: “Would we get the same result if we carried out the same experiment in a different setting, or more exactly, would the program that is being evaluated have the same effect if it were implemented elsewhere (not in the context of an experiment)?” (Banerjee and Duflo, 2009, p. 159). Although all studies have limits to generalizability, and many experiments can be replicated, many economists have asked whether low external validity of new field experimental estimates in developing countries may be of particular concern (Rodrik, 2009, Deaton, 2010, Ravallion, 2012).^24^

This section exploits the unusual history of the field experiment we study and its data. To summarize: a high-ranking state government official in Maharashtra initially indicated agreement with the original World Bank research team to conduct a randomized field experiment in three districts. Indian districts are large, on average, with populations greater than many countries. The World Bank contracted with an independent survey company to collect data in all three districts. However, the state and district governments ultimately only attempted to conduct an intervention in one district; in the other two districts, there was never any experiment.

How generalizable are the results in Ahmednagar? All empirical studies offer an imperfect combination of internal and external validity. This dataset offers a special opportunity to assess external validity directly, because of two unusual facts:

• The research decision process is recorded such that it is known which districts *could have been* part of the experimental frame, but were not.
• The same data collection mechanism that was used to construct the experimental dataset was used at the same time to collect comparable data in the non-experimental districts.

In this section, we show that the sanitation program statistically interacted with female literacy, which we interpret as a marker of greater human development more generally: the effect of latrine promotion was greatest when adult women could read. This is important because female literacy was much more common in Ahmednagar, where the program occurred, than in Nanded and Nandurbar. The average effect of the program that would be predicted by the female literacy rates in these districts may have been much smaller.

### 5.1 Heterogeneous effects: a triple interaction

If the effect of the program importantly interacts with contextual variables, then those variables may predict a different effect in different contexts. This section documents a robust statistical interaction with female literacy. To be clear, this is an *ex post* test conducted after the experiment. There is no evidence that the original World Bank research team intended to test for this interaction. We do not claim that this interaction necessarily represents a *causal* pathway, nor can we verify a mechanism through literacy *per se*, rather than some variable with which it is correlated; instead, we note that it is statistically predictive of the average treatment effect.

It is nevertheless plausible that health information and sanitation promotion could do more to promote child health and human capital accumulation where women are better educated, especially in a society where almost all childcare is done by women. Health-education gradients have been documented in many contexts (Cutler and Lleras-Muney, 2010, Vogl, 2012). The community sanitation program, in part, intended to teach village residents that feces contain microscopic germs that transmit and create preventable disease; Preston (1996) uses U.S. census data to show that mortality rates for children of schoolteachers were similar to others’ children before the germ theory of disease but fell below average as knowledge of how to protect children against germs spread in the early 20th century.

Sanitation promotion may be particularly enhanced by a context of female literacy. Mehta (2011) notes that women play an important role in Community-Led Total Sanitation programs: “ordinary women... are often the ones to persuade their husbands and families to start constructing and using a toilet” (p. 9). Mahbub (2011) reports specific examples of rural women's importance in CLTS implementation in Bangladesh. Even if they are not trying to protect their children's health, more empowered women may be more likely to promote latrine use, if only for their own well-being; it is often claimed in the Indian sanitation literature that rural Indian women bear more costs of poor sanitation than men (e.g. Alok, 2010, p. 7). Finally, in an O-ring model of health production, female literacy may merely reflect other health inputs which are complementary to reductions in the disease burden.

In Panel A of Table 7 we test for a heterogeneous effect of the program using the data from Ahmednagar district only. We report estimates of the following regression:

(4) $$z_{\mathrm{ivt}}=\beta_{1}{\mathrm{treatment}}_{v}+\beta_{2}{\mathrm{treatment}}_{v}\times {\mathrm{after}}_{t}+\beta_{3}{\mathrm{literate}\mathrm{female}}_{i}+\beta_{4}{\mathrm{treatment}}_{v}\times {\mathrm{literate}\mathrm{female}}_{i}+\beta_{5}{\mathrm{after}}_{t}\times {\mathrm{literate}\mathrm{female}}_{i}+\beta_{6}{\mathrm{treatment}}_{v}\times {\mathrm{after}}_{t}\times {\mathrm{literate}\mathrm{female}}_{i}+A_{\mathrm{ivt}}\Gamma +\gamma_{t}+{ɛ}_{\mathrm{ivt}},$$

where after_*t*_ is a property of the time period and is an indicator for being after period 1 and literatefemale_*i*_ is a property of the child's household and is an indicator for the household having a literate female adult. The coefficient on the triple interaction, *β*_6_, is the coefficient of interest. It is an estimate of the extent to which the treatment effect differed, on average, between households with and without a literate female. This will be useful as an input into our consideration of whether the program might have had different effects if it were implemented in the other districts where it was originally planned.

We report the coefficient on the triple interaction as well as the coefficient on female literacy to verify that children of female-literate households are taller, on average (our regressions include the full factorial triple interaction).^25^ In column 2, we add 8 additional controls, for the full triple interactions of the program effect with indicators that the household belongs to a Scheduled Caste and to a Scheduled Tribe; in column 3, we add 4 further controls for household electrification. Controlling for interactions with these other dimensions of socio-economic status does not change the triple interaction coefficient on female literacy. Therefore, within Ahmednagar, it robustly appears the program had a larger average effect on children living in households with a literate adult female.^26^

### 5.2 Different effects in different places?

Panel A of Table 7 documented that the effect was greater, on average, in the presence of female literacy. Panel B observes that female literacy — as measured in the same data set used to estimate program effects — is notably better in Ahmednagar than in the other two districts. About twice as many children in our data live in households with a literate adult female in Ahmednagar as in the other districts. Recall that aggregate data in Table 1 similarly showed higher female literacy in Ahmednagar, as well as lower infant mortality and a better district-level Human Development Index.

What does this contextual heterogeneity imply for the effect of the experiment? Of course, these data cannot say what the effect in fact would have been if the experiment had occurred in all three districts. Further, there is no reason to believe that female literacy is the only important difference across these districts, although it may be correlated with many of the others. However, the triple interaction can be used to linearly *predict* the average effect of the program in a district as ${\hat{\beta }}_{2}+\hat{\beta_{6}}\times \bar{{\mathrm{literate}\mathrm{female}}_{i}}$, where coefficient subscripts refer to Eq. (4). Panel C reports these predicted effects. Given the lower levels of female literacy in Nanded and Nandurbar, a much smaller average effect is predicted for these districts than for Ahmednagar. Indeed, the effect is close to zero in Nandurbar. Moreover, neither of these effects would be statistically significantly different from zero, hypothetically given the standard errors associated with the coefficient estimates in Table 5. More broadly, the corresponding fraction of rural children under five living in households with a literate adult female in all of India is 0.341, according to India's 2005 Demographic and Health Survey. This predicts an average effect of 0.149 if the experiment were conducted throughout rural India. This effect size is less than half of the effect estimated in Ahmednagar, but is closely comparable to Spears’ (2012a) estimates around 0.2 for the TSC throughout rural India.

## 6 Conclusion

We have analyzed data from a randomized controlled trial of a community sanitation program in Ahmednagar district of Maharashtra, India, as imperfectly implemented by the Government of Maharashtra. The program was associated with a 0.3–0.4 standard deviation increase in children's height-for-age *z*-scores (95% confidence interval [0.04–0.61]), or approximately 1.3 cm in a four-year old. This is comparable in magnitude to the estimates of open defecation on child height from a recent study combining data from three field experiments (Gertler et al., 2015, 0.344 to 0.460 height-for-age standard deviations), although unlike those studies ours does not observe open defecation directly.

As Section 5 explored, Ahmednagar district may have been particularly likely to show a large effect. In essentially all field experiments, non-randomized processes shape the selection of contexts and implementation partners.^27^ Note that replicating the experiment in other countries or other Indian states would not prevent any bias that might occur if less challenging environments are consistently more likely to be selected for the replications *within* each state or country. The estimated effect of the program in Ahmednagar is large; however, we use effect heterogeneity from within the experiment to predict an India-wide effect size that is roughly comparable to what Spears (2012a) estimates for the effect of the TSC on height over all of rural India. To emphasize, the fact that an effect of open defecation on child height is not *uniform* across all contexts would be no evidence that it is not an important influence on child height in many circumstances. Because much open defecation remains in rural India, this result suggests that sanitation — a classic public good — is a human development policy priority.

## Figures and Tables

**Table 1:** Comparison among studied districts.

	Source	Ahmednagar	Nanded	Nandurbar	Rural Maharashtra	Rural India
Population, millions	2001 census	4.1	2.7	1.3	41.1	742.5
Population, millions	2011 census	4.5	3.4	1.3		833.4
Urban population %	2001 census	19.9	24.0	15.4	42.4^a^	27.8^a^
Population density (per km^2^)	2001 census	240	260	220	181–314	230–312
Scheduled Tribe %	2008 DLHS	12.7	16.9	71.4	23.6	23.1
Scheduled Tribe %	2001 census	7.5	8.8	65.3		
Scheduled Caste %	2001 census	12.0	17.3	3.2		

Infant mortality rate (per 1000)	2001 census	44	61	64	53	73
Open defecation %	2011 census	48.7	65.6	65.4	62.0	69.3
With toilet facility %	2008 DLHS	52.3	31.1	19.6	32.5	34.2
Open defecation %	2001 census				81.8	78.1

Human development index	2000 SHDR	0.57	0.36	0.20		
Rural female literacy	2001 census	61.4	49.9	40.2		
Rural male literacy	2011 census	67.9	62.1	51.5		
Electricity %	2011 census	75.1	74.5	58.3	73.8	55.3
Modern housing materials %	2008 DLHS	39.3	50.4	7.3	16.8	19.6

DLHS is the Indian government's District Level Health Survey. SHDR is the Maharashtra state human development report.

a Fraction of all of population of Maharashtra and India that live in urban areas.

**Table 2:** Balance of baseline sample means.

	Ahmednagar district			Nanded and Nandurbar		
	Control	Treatment	*t*	Planned control	Planned treatment	*t*
Height for age	−2.58	−2.68	−0.82	−3.70	−3.66	0.24
Has vaccine card	0.95	0.94	−0.46	0.86	0.81	−1.46
Fed breastmilk at birth	0.98	0.99	0.74	0.97	0.97	−0.13
Months exclusively breastfed	4.80	5.21	1.09	5.75	5.95	1.10
Total months breastfed	7.57	8.03	0.59	9.99	10.67	1.22
Female	0.46	0.51	1.38	0.52	0.50	−0.95
Age in months	37.76	37.37	−0.37	38.84	39.26	0.61
Asset index 1 (first component)	−0.72	−1.03	−1.30	0.41	0.47	0.38
Asset index 2 (second component)	0.06	0.06	0.01	−0.03	−0.03	−0.06
Owns toilet or latrine	0.10	0.18	1.47	0.05	0.06	0.68
Owns separate kitchen	0.62	0.65	1.03	0.48	0.44	−1.52
Owns clock or watch	0.73	0.74	0.39	0.51	0.51	−0.09
Adult female literacy	0.50	0.52	−0.46	0.28	0.28	−0.29
Adult literacy	0.62	0.64	−0.74	0.41	0.41	−0.12
						

Count of survey rounds in which measured	1.91	1.94	0.71	2.02	2.05	0.91
Measured in all three rounds	0.22	0.22	0.13	0.28	0.31	1.26

*n* (children under 5)	1686	1754		3967	3953	
Villages	30	30		60	60	

**Table 3:** Distribution of sample and ages across survey rounds, Ahmednagar.

	Round 1	Round 2	Round 3
*Panel A: Sample count (total n* = *3432)*			
Appears once	234	339	444
Appears twice	390	742	512
Appears three times	257	257	257

Total	881	1338	1,213

*Panel B: Average age in months (mean: 32.9)*			
Appears once	43.8	31.7	19.4
Appears twice	39.1	30.5	34.7
Appears three times	29.6	32.7	45.3

Sample corresponds with height sample from panels A and B of main results Table 5.

**Table 4:** Effects of program on sanitation are comparable to modest effects in the literature

	(1)	(2)	(3)
	Control	Treatment	Difference
*Panel A: Evidence of effect of experiment on sanitation in Ahmednagar*			
Households reporting recollection of TSC sanitation promotion visit, midline	0.285	0.357	0.072†
			(0.043)
Household latrine ownership, endline	0.146	0.228	0.081†
			(0.045)
won Clean Village Prize for elimination of open defecation 2006–2012	0.100	0.300	0.200†
	(3 of 30)	(9 of 30)	(0.102)

*Panel B: Effects of promotion interventions on sanitation in the literature*			
Effect on latrine ownership in Orissa of TSC information campaign (Pattanayak et al., 2009)	0.13	0.32	0.190
			(*p* = 0.006)
Effect on latrine ownership in Haryana of “no toilet, no bride” (Stopnitzky, 2011)			0.043
			(0.007)
Effect of sanitation experiment in Indonesia on toilet construction (Cameron et al., 2013)	0.130	0.159	0.030
			(*p* = 0.072)
Effect of sanitation experiment in Indonesia on open defecation (Cameron et al., 2013)	0.532	0.488	−0.044
			(*p* = 0.025)
Effect of sanitation experiment in Madhya Pradesh on owning improved toilet (Patil et al., 2013)	0.22		0.178
	(0.01)		(0.035)
Effect of sanitation experiment in Madhya Pradesh on observed toilet use (Patil et al., 2013)	0.17		0.104
	(0.01)		(0.029)
Latrine ownership in rural Orissa after TSC [treatment only] (Barnard et al., 2013)		0.72	
Latrine use in rural Orissa after TSC [treatment only] (Barnard et al., 2013)		0.44	

Standard errors clustered by village in Panel A. Two-sided *p* values in Panel A: * *p* < 0.05.

† *p* < 0.10.

**Table 5:** Effects of the experimental program on height-for-age in Ahmednagar.

	(1)	(2)	(3)		(1)	(2)	(3)
Round × dist FEs	✓	✓	✓	Round × dist FEs	✓	✓	✓
Age × sex		✓	✓	Age × sex		✓	✓
Village FEs			✓	Village FEs			✓
*Panel A: Double difference, midline and endline*				*Panel B: Double difference, before and after*			
treatment	−0.105	−0.0988		treatment	−0.105	−0.0992	
	(0.129)	(0.129)			(0.129)	(0.129)	
treatment × midline	0.278†	0.236†	0.274*				
	(0.154)	(0.140)	(0.136)				
treatment × endline	0.379†	0.418*	0.448*	treatment × mid. or end.	0.326*	0.324*	0.357*
	(0.211)	(0.195)	(0.190)		(0.160)	(0.146)	(0.141)

*n* (children)	3432	3432	3432	*n* (children)	3432	3432	3432

*Panel C: Triple difference, midline and endline*				*Panel D: Triple difference, before and after*			
treatment	0.0412	0.0501		treatment	0.0412	0.0500	
	(0.172)	(0.172)			(0.172)	(0.172)	
treatment × Ahm. × midline	0.298	0.224	0.250				
	(0.237)	(0.232)	(0.227)				
treatment × Ahm. × endline	0.572*	0.609*	0.646*	treatment × Ahm. × mid. or end.	0.431†	0.411†	0.443*
	(0.264)	(0.256)	(0.249)		(0.226)	(0.220)	(0.213)
treatment × Ahmednagar	−0.147	−0.114		treatment × Ahmednagar	−0.147	−0.114	
	(0.214)	(0.212)			(0.214)	(0.212)	
treatment × midline	−0.0200	−0.000374	0.0154	treatment × mid. or end.	−0.105	−0.104	−0.101
	(0.181)	(0.181)	(0.178)		(0.160)	(0.161)	(0.158)
treatment × endline	−0.192	−0.211	−0.220				
	(0.160)	(0.160)	(0.158)				

*n* (children)	11,337	11,337	11,337	*n* (children)	11,337	11,337	11,337

Standard errors clustered by village. Two-sided *p* values.

Panels A and B include only Ahmednagar; panels C and D also include Nanded and Nandurbar.

† *p* < 0.10.

* *p* < 0.05.

**Table 6:** Within-child effects on growth trajectories in Ahmednagar.

	(1)	(2)	(3)	(4)	(5)
Sample:	Full	Appears 3 times	Appears 3 times	Appears 2 or 3 times	Appears 2 or 3 times
treatment	−0.105	−0.349		−0.210	
	(0.129)	(0.258)		(0.148)	
treatment × midline	0.278†	0.393	0.393	0.448*	0.284†
	(0.154)	(0.256)	(0.256)	(0.170)	(0.153)
treatment × endline	0.379†	0.493†	0.493†	0.671*	0.462*
	(0.211)	(0.272)	(0.272)	(0.204)	(0.206)
Child fixed effects			✓		✓

*n* (children under 5)	3432	771	771	2415	2415

Standard errors clustered by village. Two-sided *p*-values:

Column 1 matches column 1 of panel A of Table 5.

† *p* < 0.10.

* *p* < 0.05.

**Table 7:** External validity?: Heterogeneity of predicted effect on height-for-age by district

	(1)	(2)	(3)
*Panel A: Female literacy triple interaction regression results, within Ahmednagar*			
Literate adult female × after × treatment	0.816†	0.962*	0.920†
	(0.445)	(0.439)	(0.462)
Triple interaction *F*-test	*F*_4,59_ = 3.89	*F*_4,59_ = 3.59	*F*_4,59_ = 2.29
	*p* = 0.007	*p* = 0.011	*p* = 0.070
Literate adult female	0.483*	0.531*	0.492*
	(0.208)	(0.208)	(0.224)
Literacy full triple interaction	✓	✓	✓
Survey round fixed effects	✓	✓	✓
SC and ST triple full interactions		✓	✓
Electrification full triple interaction			✓
*n*	3,047	3,047	3,047

*Panel B: Female literacy is highest in Ahmednagar*			
District	Ahmednagar	Nanded	Nandurbar
Female literacy mean	0.621	0.355	0.207
Standard error of the mean	(0.025)	(0.026)	(0.021)
Test different from Ahmednagar		*t* =−7.34	*t* =−12.63

*Panel C: Predicted effect of sanitation intervention is greatest in Ahmednagar*			
District	Ahmednagar	Nanded	Nandurbar
Estimated observed mean effect	0.326		
	(0.160)		
Effect predicted by female literacy	0.377	0.159	0.039

Standard errors clustered by village. Two-sided *p* values.

The triple interaction *F*-test tests whether literate female, literate female × after, literate female × treatment, and literate female × after × treatment are jointly significant; these four terms are what is meant by the “full triple interaction.” The predicted effect is computed as the coefficient on treatment × after plus the product of the average female literacy rate from Panel B and the coefficient on literate female × after × treatment, with both coefficients from the regression reported in column 1 of Panel A. In panel C, “estimated observed mean effect” is from column 1 of Panel B of Table 5.

† *p* < 0.10.

* *p* < 0.05.

## Footnotes

1 Environmental enteric disfunction would be caused by repeated fecal contamination which, through an inflammatory response, increases the small intestine's permeability to pathogens while reducing nutrient absorption. This inflammation is hypothesized to have direct effects on growth-regulating hormones (Prendergast et al., 2014). If so, such inflammation could cause apparent malnutrition of various forms, stunting, and cognitive deficits, even without necessarily manifesting as diarrhea or otherwise observable illness (*see also*Petri et al., 2008, Mondal et al., 2012, Korpe and Petri, 2012). Lin et al. (2013) show that children in Bangladesh who are exposed to more fecal environmental contamination are more likely to exhibit biological markers of enteropathy, and in turn suffer impaired growth. In longitudinal data from field sites in eight countries, Kosek et al. (2013) show that environmental enteropathy is associated with subsequent deficits in growth.

2 It is not the purpose of this paper to conduct a “program evaluation” of the TSC overall; for an impact evaluation demonstrating that the TSC overall improved infant mortality and child height, on average, see (Spears, 2012a). For a study of the India-wide average effect on later-childhood cognitive achievement of exposure to the first years of the TSC in early life, see Spears and Lamba (2015). Instead, this paper uses a modest experimental implementation of the TSC in one district to learn about the effect of sanitation on early-life human capital accumulation. Thus, whenever this paper refers to “the program” studied, we mean only this special, randomized sanitation promotion intervention.

3 For more details on the exact procedures of a sanitation “triggering,” please see Bongartz and Chambers (2009). The program studied was not a traditional CLTS implementation because it also included government subsidies for latrine construction which traditional CLTS forbids. A member of the World Bank team that oversaw the program writes that “each [village] was assigned to an extension worker with the [rural district government], such as a teacher, agricultural extension worker, health worker … along with a supervisor from the block level. These motivators visited villages and undertook initial triggering activities and follow-up activities which would include participatory approaches (e.g. CLTS methods), individual, small or large group meetings, visits to nearby villages which had demonstrated local action, etc. There was extensive follow-up for demand creation, followed by inputs on technology [latrine] options.”

4 The upper bound on a 95% confidence interval for the effect on child height is 0.127. Because the intervention caused a decline in open defecation of only 9 percentage points, these results suggest that the large confidence interval of an instrumental variables estimate of the effect of local open defecation on child height-for-age would include zero *and* estimate in the range that we document.

5 In Ahmednagar, female literacy is highest, and infant mortality is lowest; the 2000 state human development report gave Ahmednagar the highest district-level human development index. These three districts were initially chosen for the experiment in discussions between the government of Maharashtra and the original World Bank research team because Ahmednagar and Nanded district officials requested early implementation of the TSC at a state-level workshop in 2002. A senior state official selected Nandurbar so that a particularly poor and challenging district would be included. Randomization would occur separately stratified within each district to assign 60 villages to treatment and control groups, with 30 villages each in each district.

6 Indian districts are large, with a population of 4.5 million people; Ahmednagar district is larger than many *countries*, including similarly-sized New Zealand, Liberia, and Uruguay.

7 This randomization was done once (that is, without rebalancing on covariates), by the original World Bank research team, in an independent Excel spreadsheet for Ahmednagar district.

8 Among recorded children whose heights should have been measured, 12.6% of children are recorded by surveyors as not having had height measured in the household interview because they were “out of station” and 5.1% are recorded as “not present at home;” extant documentation does not clarify how these categories are different. Balance Table 2 confirms that attrition (and appearance across survey rounds more generally) was balanced across treatment and control groups.

9 An indicator for height-for-age below −2.

10 For example, applying zscore06 perfectly replicates the transformation from raw height to *z*-scores in India's Rapid Survey of Children data.

11 Height-for-age is declining, on average, over the first two years of life in India and other developing countries; in addition to improving model fit, these controls rule out any spurious effect of differences in village age structure.

12 The two-sided *p*-value differs across household-level regressions with standard errors clustered by village (0.116), regressions collapsed to 60 village-mean observations (0.099), and in a non-parametric Mann–Whitney–Wilcoxon test on the collapsed means (0.066).

13 In rural India, latrine ownership does not imply latrine use; it is very common, for example, for only young adult women (such as new daughters-in-law) or the very old or disabled to use latrines, while older women and all males bypass even functioning latrines to defecate in the open (Coffey et al., 2014).

14 With robust regression *t*, Mann–Whitney, and Fisher exact tests with *p*-values of 0.054, 0.055, and 0.052, respectively.

15 We note that a widely circulated working paper version of this paper incorrectly claimed that these data were not collected; we mistakenly believed this on the basis of the then-available data that had survived from the 2004 experiment, but more data have subsequently been found. We regret this error. It is nevertheless correct that original research documents emphasized child height as the unique intended outcome. Despite the fact that many epidemiological studies use survey-reported diarrhea as a dependent variable, the original World Bank research team noted that reported diarrhea is recognized as a noisy and unreliable outcome measure (Schmidt et al., 2011). For example, Zwane et al. (2011) show that households randomly selected to be surveyed more frequently report less child diarrhea. More broadly about survey reported morbidity, in a survey experiment in India, Das et al. (2012) find that changing the recall period reverses the sign of the apparent healthcare–economic status gradient. Finally, although not widely recognized at the time of the original survey in 2004, Humphrey's (2009) proposal of height shortfalls due to chronic enteropathy suggests that diarrhea may not be an indicator of or necessary condition for losses in human capital.

16 Non-parametric statistical significance tests, collapsed to the village level, further confirm these findings. Twice, once for the midline and once for the endline, we create a dataset of 60 observations: for each village we compute first the mean height-for-age *z*-score in each round, and then the change since the first round. We perform a Mann–Whitney–Wilcoxon rank sum test. The null hypothesis that the distributions from which the changes in mean height were drawn were the same in treatment and control villages are almost rejected in the baseline-to-midline case with a two-sided *p*-value of 0.103 and is rejected in the baseline-to-endline case with a *p*-value of 0.065. Repeating this procedure a third time with the midline and endline collapsed into a single “after” period produces a *p*-value of 0.048.

17 Note that although this resembles a growth curve, it is from a *synthetic* cohort – that is to say, a cross-section – and does not plot the longitudinal growth of any child.

18 We collapse the data into 240 observations: separate means for boys and girls of each age, in the treatment and control groups. A matched pairs test rejects no effect with a *p*-value is 0.039. Because this test compares children within age-in-months by sex categories, it also is unaffected by any concern that the WHO reference population may not be appropriate, due to, say, age or gender bias relative to international standards (Barcellos et al., 2014).

19 In such a panel, initially 4.5-year-old children would be measured again twice, even though one might not expect a large effect of open defecation on their already-determined height trajectory, even if there were large effects on still-malleable younger children; this would bias estimates towards zero.

20 For the means (that is, the regression coefficients), child fixed effects are absorbed by the village fixed effects and are perfectly correlated with treatment; for variances, estimated standard errors do not change because they are clustered at the village level, which is coarser.

21 The subset is children who live in households who did not have a latrine at endline; this therefore excludes children who live in households who did not have a latrine at baseline but who acquired one by endline.

22 Assuming that the treatment never perversely caused any household to abandon latrine ownership.

23 In the control group, the standard deviation of the within-child change in child height from the baseline to the endline is only 1.78 *z*-score points.

24 For example, Allcott and Mullainathan (2012) show that different implementing organizations in an experimental energy conservation program in the U.S. produce predictably different effects, and that implementing organizations are, in practice, chosen according to characteristics correlated with treatment effects. Our paper, in contrast, studies implementation by the Indian government, but focuses on the threat most emphasized in development economics: heterogeneity in program effects across different populations and geographic contexts.

25 The table is compressed here to save space; a reader interested in all coefficients from the triple interaction in all three models may see Table A5 in the Online Supplementary Appendix.

26 Section 4.5.3 reported evidence of negative externalities: the effect was found even in households that did not have a latrine in the endline survey. This is true of the triple difference, as well, which remains similar at 0.873 (*t* = 1.68) when the sample is restricted to households without latrines.

27 As an anonymous reviewer of this paper suggested: “This kind of back-of-the envelope calculation can probably be done in most RCTs when there are significant interactions between treatment effects and commonly observed characteristics of the population.” Indeed perhaps it should be, if there are reasons to believe these characteristics are unusual.
