# Supplementary material for: Village sanitation and child health: Effects and external validity in a randomized field experiment in rural India
Source: J Health Econ. 2016 Jul;48:135–48. doi: 10.1016/j.jhealeco.2016.03.003 (PMC4920645; doi:10.1016/j.jhealeco.2016.03.003)
Supplement: Supplementary file 1 [file mmc1.pdf]

Supplementary appendix A to

Village sanitation and child health:
Evidence from a randomized field experiment in rural India

A1. No evidence of an effect of the experiment on health beliefs

	(1)	(2)	(3)	(4)	(5)
**question:**	prevent diarrhea	prevent diarrhea	prevent diarrhea	treat diarrhea	treat diarrhea
**response:**	latrine use	clean water	don't know	fluids/ORS	don't know
**treatment village**	-0.00472	0.0260	-0.0236	0.0260	-0.00830
	(0.00377)	(0.0320)	(0.0175)	(0.0436)	(0.00716)
**constant (control mean)**	0.00988**	0.788**	0.0791**	0.280**	0.0309**
	(0.00330)	(0.0221)	(0.0120)	(0.0290)	(0.00493)
**household respondents**	3,169	3,169	3,169	3,169	3,169

The table above presents linear probability regressions of an indicator for the listed response for each question, on an indicator variable for being in the treatment group, for Ahmednagar district in the endline survey. Respondents in the treatment group were not more likely to answer these questions correctly. Consistently with the program design, this suggests that the health effects of the program were not through effects of the latrine promotion program on health beliefs (rather than sanitation behavior).

A2. “Dose-response” externalities graph: children in households with or without a latrine are taller, on average, in villages where more of their neighbors have a latrine

Ahmednagar, midline and endline survey:

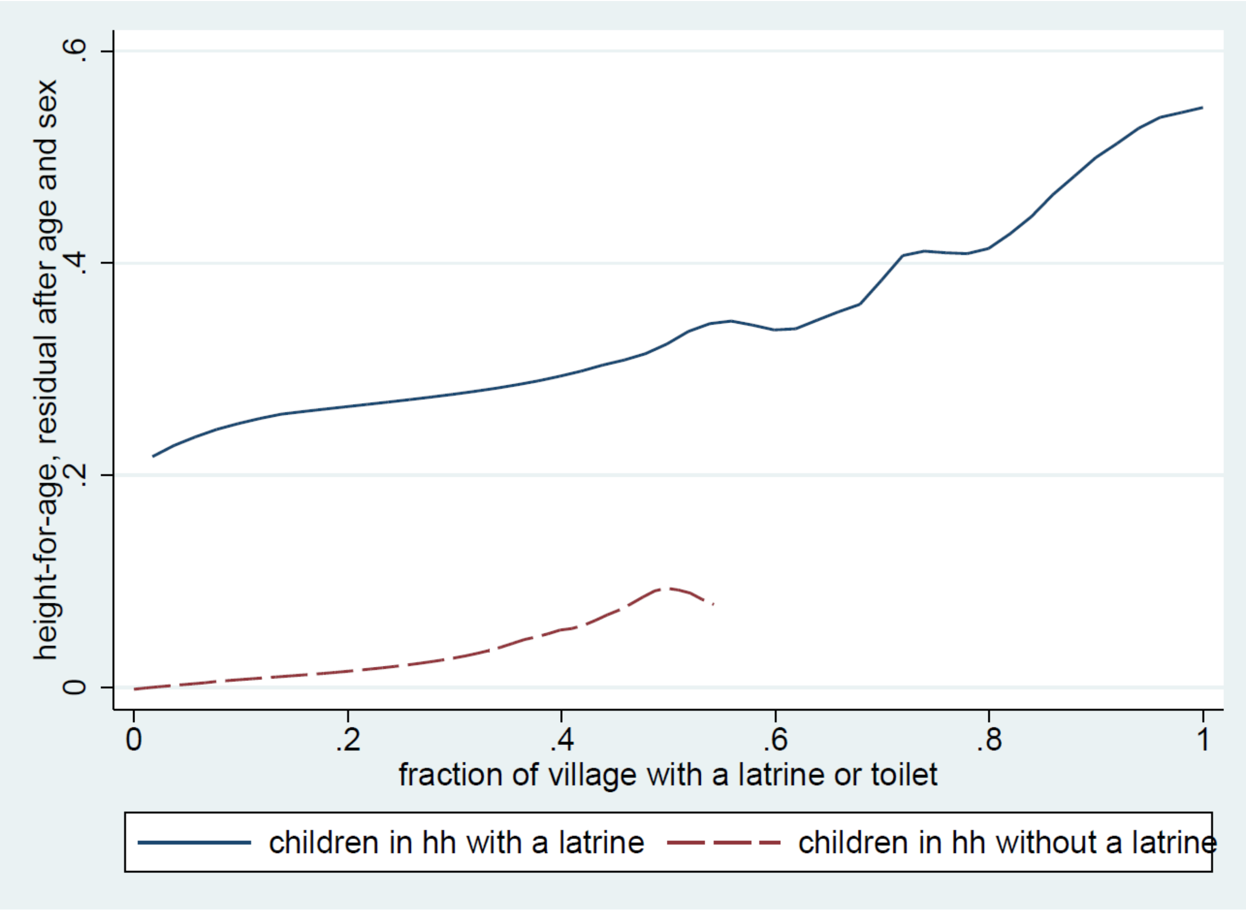

Using only data from the endline survey in Ahmednagar, and controlling for a child’s age in months and sex and whether her own household has a latrine, village latrine coverage is associated with child height: a child living in a village with full latrine coverage is 0.56 height-for-age standard deviations taller, on average, than a child in a village where no households have a latrine (*p*= 0.074). This linear partial correlation is certainly not an estimate of a causal effect, but is quantitatively comparable with other estimates in the literature.

A3. Dichotomized stunting

Spears, et al. (2013) report Monte Carlo simulations showing that using dichotomized stunting as a dependent variable, rather than continuous height-for-age, sacrifices statistical power (much like using dichotomized poverty indicators rather than a continuous measure of income or consumption). Nevertheless, we report results for dichotomized stunting ($z \leq -2$) because this measure is commonly used, especially in the clinical literature.

55% of the 3,432 observations in our main height sample for Ahmednagar are stunted, an internationally high figure that is unfortunately unsurprising for rural India. The table below presents regression results comparable to Panel A of Table 5 in our main results.

	(1)	(2)
	stunted	stunted
sample:	full	longitudinal
treatment	-0.0127	0.0294
	(0.0348)	(0.0391)
treatment × midline	-0.00442	-0.0246
	(0.0393)	(0.0445)
treatment × endline	-0.0699	-0.146*
	(0.0488)	(0.0564)
n	3432	2415

Clustered standard errors in parentheses
+ p<0.1, * p<0.05

The "longitudinal" sample in column 2 is the within-child sample corresponding to columns 4 and 5 of table 6.

A4. Robustness to alternative height-for-age truncation cut-points

Height-for-age *z*-scores, especially for young children, require accurately measured age in months. Mismeasuring ages (as well as mismeasuring heights) will add noise to the *z*-scores. Thus, in our data, the standard deviation of scaled height-for-age is 2.1, more than twice as much as would be expected from a standardized normal distribution. Supplementary Appendix B is devoted to analysis of the possible consequences of this dispersion.

One consequence of this noise is to reduce power by increasing standard errors. Another is to require cut-points beyond which data are omitted.

Could this truncation be responsible for our results? Would other endpoints produce different answers? In order to answer this question, the table on the next page reports results from 49 alternative combinations of endpoints, in 0.5 standard deviation increments of a lower bound of -9 to -6 and an upper bound of 3 to 6. In particular, the table replicates column 2 of panel B of table 5, using the double-difference with a collapsed “after” so that there is only one treatment effect estimate to report for different combinations.

Changing the cut-points has little effect on the result. All coefficient estimates are positive, and 94 percent (all but the extreme lower-right corner) are between 0.2 and 0.4 height-for-age standard deviations, comparable to the range in table 5. The bottom panel reports corresponding *t*-statistics; most exceed 2, especially near the cutpoints that we use. Unsurprisingly, estimates become less precise as the bounds are widened and noisier observations are included. The mean across all combinations of cut-points is an effect of 0.3 standard deviations and a *t*-statistic of 1.96.

An alternative approach is to use log of height as the dependent variable, omitting *z*-scores altogether. If this is done with the widest set of cutpoints used in the table, -9 to 6, we find that the program increased height in the endline by 3.3 percent with a *t*-statistic of 2.42.

Panel A: Coefficients on treatment × after

lower limit	upper limit on height for age z-scores 3.0	4.0	4.5	5.0	5.5	6.0	mean
-9.0	0.336	0.328	0.333	0.301	0.296	0.252	0.309
-8.5	0.329	0.320	0.325	0.292	0.287	0.242	0.301
-8.0	0.365	0.356	0.361	0.331	0.326	0.281	0.338
-7.5	0.345	0.336	0.341	0.311	0.306	0.261	0.318
-7.0	0.327	0.318	0.324	0.294	0.290	0.244	0.301
-6.5	0.331	0.321	0.327	0.296	0.291	0.245	0.303
-6.0	0.229	0.218	0.226	0.193	0.188	0.142	0.201
mean	0.323	0.314	0.319	0.288	0.283	0.238	0.296

Panel B: t-statistics on treatment × after

lower limit	upper limit on height for age z-scores 3.0	4.0	4.5	5.0	5.5	6.0	mean
-9.0	2.05	1.99	2.04	1.70	1.63	1.36	1.82
-8.5	2.17	2.10	2.14	1.77	1.69	1.39	1.91
-8.0	2.62	2.52	2.55	2.15	2.06	1.73	2.31
-7.5	2.44	2.36	2.38	2.01	1.91	1.58	2.15
-7.0	2.36	2.25	2.29	1.89	1.80	1.48	2.04
-6.5	2.41	2.26	2.29	1.88	1.79	1.47	2.05
-6.0	1.73	1.59	1.64	1.28	1.21	0.90	1.42
mean	2.25	2.15	2.19	1.81	1.73	1.42	1.96

A5. Complete triple interaction table

Panel A of table 7 suppresses the full triple interaction so that the table can fit on one page, along with the information about the external validity prediction. Here, we report the full set of coefficients from the triple interactions in the table:

	(1)	(2)	(3)
		height-for-age z-score	
treatment	0.188	0.326	0.389
	(0.274)	(0.293)	(0.342)
treatment × after	-0.130	-0.389	-0.413
	(0.338)	(0.343)	(0.352)
round dummies	✓	✓	✓
sex × age	✓	✓	✓
**treatment × after × fem lit**	**0.816+**	**0.962***	**0.920+**
	**(0.445)**	**(0.439)**	**(0.462)**
female literacy	0.483*	0.531*	0.492*
	(0.208)	(0.208)	(0.224)
treatment × fem. lit.	-0.500	-0.575+	-0.556
	(0.345)	(0.332)	(0.344)
after × fem. lit.	-0.261	-0.393	-0.455
	(0.289)	(0.288)	(0.321)
ST triple interaction		✓	✓
SC triple interaction		✓	✓
electricity triple interaction			✓
constant	-2.693**	-2.806**	-2.896**
	(0.213)	(0.235)	(0.258)
n	3047	3047	3047
