# Supplementary material for: Village sanitation and child health: Effects and external validity in a randomized field experiment in rural India
Source: J Health Econ. 2016 Jul;48:135–48. doi: 10.1016/j.jhealeco.2016.03.003 (PMC4920645; doi:10.1016/j.jhealeco.2016.03.003)
Supplement: Supplementary file 2 [file mmc2.pdf]

SUPPLEMENTARY APPENDIX B: THE DISTRIBUTION OF HEIGHT-FOR-AGE IN OUR SAMPLE, AND CONSEQUENCES OF LOW VALUES

B1. Introduction

The height-for-age z-score data used in our main analysis is more widely dispersed than the WHO reference sample: many children in our sample from rural India fall below the cutpoint of even -6 standard deviations below the mean. This is one reason that our analysis replicates its results using log of height in centimeters and dichotomized stunting as dependent variables.

Is the dispersion — and presence of extreme negative values – evidence against the credibility of our data or our results? In this supplementary analysis, we provide three pieces of evidence relevant to the dispersion of our data:

- **Comparability with DHS and IHDS.** First, in section B2, we compare the dispersion of our data and the fraction of children whose measured height is very short with two other widely used data sources: the DHS and the IHDS. In so doing, we consider the role of `zscore06`, the standard and widely-used Stata command we use to compute z-scores. In the DHS, we find that a considerable number of children with height for age less than -6 are found when this command is used to compute height-for-age from raw height. We further find that the dispersion in our data – and fraction of extremely short heights – is less in our data than in the IHDS, a widely used nationally representative dataset on the heights of children in India. These facts may suggest that our sample of heights is not prima facie impossible or erroneous.
- **Robustness to omitting extreme or influential observations.** Second, in section B3, we demonstrate that our result is not driven by a few apparently extremely short children, or by an otherwise influential small number of outliers. Four different methods of identifying potentially questionable observations all agree that the result is robust to such observations' exclusion.
- **An alternative truncation sample.** Finally, in section B4, we show that our results are qualitatively similar in the most trustworthy specifications if alternative cutpoints are used that discard children whose heights are below the DHS India-wide truncation point of -6.

B2. Comparison with other data sources

B2.1. **Demographic and Health Survey.** One important source of data on child height is the Demographic and Health Survey (DHS). The most recent DHS in India was conducted in 2005-6, approximately the same time as our experiment. The height for age z-scores included in the publicly available DHS data set are truncated at -6 and 6. Therefore, any paper that uses these included z-scores is constrained to this truncation, whether or not it is appropriate for that analysis.

In our analysis, we computed z-scores using the Stata user-written command `zscore06` by Jef Leroy. This software constitutes a standard approach to the computation of z-scores in health economics; it is widely downloaded, used, and cited. For example, the Government of India's recent Rapid Survey of Children includes z-scores which exactly match those yielded if `zscore06` is applied to its raw survey data.

When `zscore06` is applied to the height, age in months, and sex data in the Indian DHS, the computed z-scores are very similar but not identical to those included in the DHS.[1] 5.6% of children under 5 in the DHS with sufficiently complete information to compute a height-for-age z-score nevertheless do not have one reported within the DHS data. Among children with a height-for-age z score included in the DHS, the included scores have an R^2 of 98% when regressed on scores computed with `zscore06`.

What is relevant for our analysis is that even among the exact same sample of children with DHS z-scores, the dispersion of z-scores is slightly greater for scores computed with `zscore06` than for the scores included in the DHS. Moreover, among the rural DHS sample – which is the relevant comparison group for the all-rural sample in our experiment – fully 2.4% of observations have a height for age z-score below -6 as computed by `zscore06`. This is considerably more than the 0.6% of rural observations that would be predicted to be below -6 height-for-age standard deviations by applying the mean and standard deviation of the scores included in the DHS to a normal distribution. However, this figure is comparable to the 3.8% of observations in the sample from our experiment that we use in our main analysis.

Therefore, applying the same standard method of computing z-scores as is used in our analysis to the DHS, a data source that is widely considered to be of relatively high quality for econometric analysis of child height, we find that a non-trivial fraction of observations present z-scores below -6. This finding suggests that it is not the case that a survey of rural India should expect to find zero children of height-for-age below -6; although we do not believe that the data that we use is as high quality as is the DHS, these statistics provide no evidence that our data is of sufficiently poor quality to be incredible.

Note that both in the main text of our paper and later in this supplementary appendix we additionally present results using the log of height in centimeters as a dependent variable, rather than height-for-age z-scores. Such a method assumes that the effect of the program is a proportional percent (entering height in centimeters linearly would ignore the fact that a, say, 2 centimeter increase is different for a four year old and a four month old). The robustness of our result to this change indicates that neither z-scores in general nor the `zscore06` software is responsible for our findings.

B2.2. **India Human Development Survey.** The India Human Development Survey (IHDS) collected data on the height of children under 5 years old in an NIH-supported nationally representative sample of Indian households. Data were collected in two waves: a first wave in 2005 and a second wave in 2012 that is not publicly available. We were provided special access to this data by the team that collected it for the purpose of assessing the quality of the height data. We

[1] At the end of this appendix, we include a Stata log file as a supplementary attachment to our paper, documenting this computation. Because our paper does not use DHS data, it is beyond the scope of this note to reconstruct how z-scores were computed in the DHS.

Table B1. Dispersion of height-for-age in our data and in the IHDS

	all observations	rural observations		rural, between -8 and 4	
	std. dev.	below -6	fraction out	mean	std. dev.
our data	3.823	0.113	0.098	-2.334	1.919
IHDS-I 2005	3.793	0.214	0.118	-3.272	2.241
IHDS-II 2012	4.756	0.157	0.115	-2.71	2.155

are grateful to the University of Maryland and NCAER research team for sharing these data.

If the IHDS contains more dispersed data, and specifically if the IHDS contains more children with extremely negative height-for-age scores, then this would be evidence that such observed heights are not impossible and that the quality of the data we study is not implausibly low. Additionally, Spears (2012) reports that height is particularly correlated with cognitive achievement in India (specifically by more than in the United States), using the exact IHDS-2005 height-for-age data used here; therefore, dispersion in height in the IHDS is not mere noise.

In table B1, we find that height-for-age of comparable children is more dispersed in the IHDS than in our main data (Ahmednagar district, rounds 1-3, treatment and control groups), by a variety of metrics. A larger fraction of the data is below the -6 cutpoint used by the IHDS and a larger fraction is outside of the -8 to -4 range used in the main sample of our analysis. Within this range, the standard deviation of height-for-age is greater in the IHDS than in our data.

As discussed above, the DHS only releases its computed z-scores for children whose height-for-age is between -6 and 6. The mean and standard deviation of the DHS data is -1.85 and 1.67 for children in rural India using their computations, or -1.92 and 1.68 using `zscore06`. Because of the different selection of cutpoints, the DHS must have a greater mean, but we note that the standard deviation in our -8 to -4 data is closer to the DHS standard deviation than the IHDS standard deviation in the same sample is to the DHS.

Figure B1 presents more evidence on the comparison between the IHDS and our data, using estimated kernel densities. The graph truncates the data at the -8 to +4 range used in our main analyses: notice that the density of our data is very close to 0 at these cutpoints. In contrast, the density of the IHDS is visibly above 0 even at -8: it includes many more very short children than does our data.

B3. Extreme values are not responsible for our result

If our results were merely driven by apparent observations of extremely short children which are in fact not trustworthy data, then we would expect an effect of the program not to be found when such outliers are omitted from the analysis. In this section, we consider three separate methods of identifying outliers and find that the results are robust to their exclusion in each case.

B3.1. **Omitting influential observations.** Cook's D and leverage are two standard measures of the influence of regression observations, used to identify outliers. Figure B2 documents that our result is robust to the exclusion of influential observations by both of these measures. Presented are point estimates and confidence

FIGURE B1. Kernel density of height-for-age in our data and in the IHDS

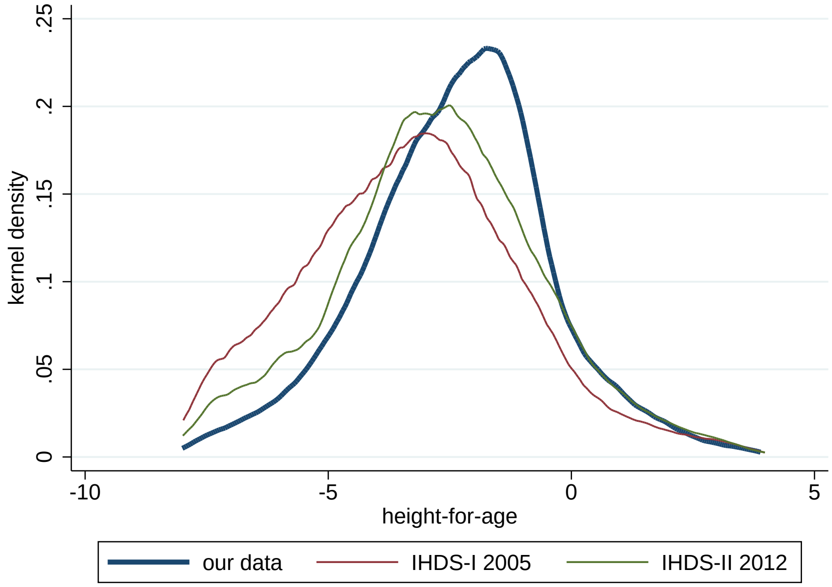

intervals equivalent to the main result coefficient on the interaction between treatment and period 3, comparable to column 2 of panel A of table 5 of the results of the main paper.

To construct each panel, observations were sorted in decreasing order of the measure of extremeness; moving right a larger set of observations was excluded. So, for example, in the top graph the point 1 plots the confidence interval when only the observation with the largest Cook's D is excluded, and point 150 along the horizontal axis plots the confidence interval for the regression coefficient obtained when the 150 observations with the greatest Cook's Ds are excluded. The vertical line in panel (a) corresponds to the threshold of $4/n$, where n is the number of observations. Because there are 3,432 observations in the full sample, omitting the 250 most extreme values (the furthest extent of graph) is omitting 7.3% of the sample. As the flat profile of both panels shows, with either measure of extremeness, omitting this large and most influential section of the sample does not change the result.

B3.2. **Omitting very short or tall children.**

B3.2.1. *Dropping the shortest children within each age-sex bin.* One particular concern is that the height data in our sample is not merely *dispersed*: in particular many children are *short*. Could the apparently very short observations in our sample be responsible for our results? Figure B3 presents results from one approach to omitting the shortest children. As in figure B2, the vertical axes present confidence intervals for our main result: the interaction of treatment and period 3. The horizontal axis records how many observations are omitted from each of the 120 age and sex categories. For example, at the point marked 2, the shortest two children are omitted from each combination of sex and age-in-months. This means that, at

Figure B2. Effect estimate is robust to omitting influential observations

(a) Omitting results with high Cook's D

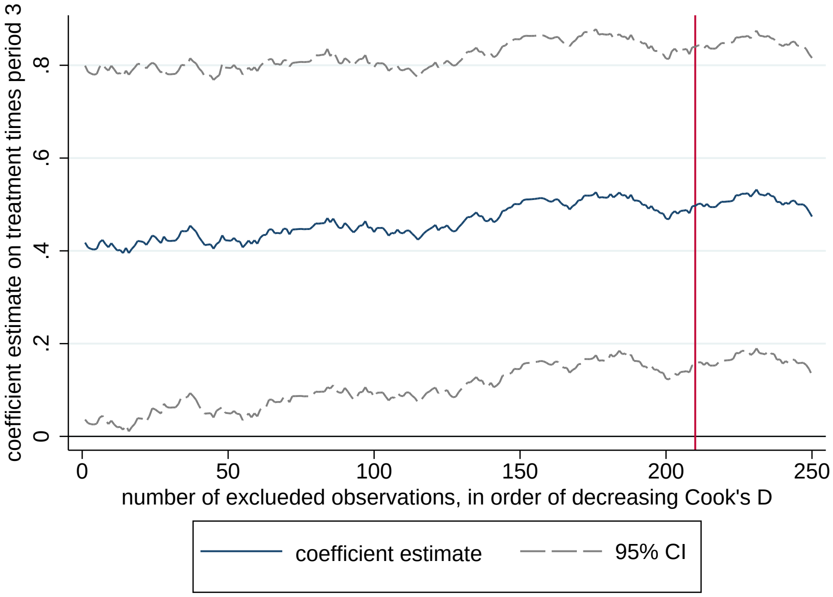

(b) Omitting results with high leverage

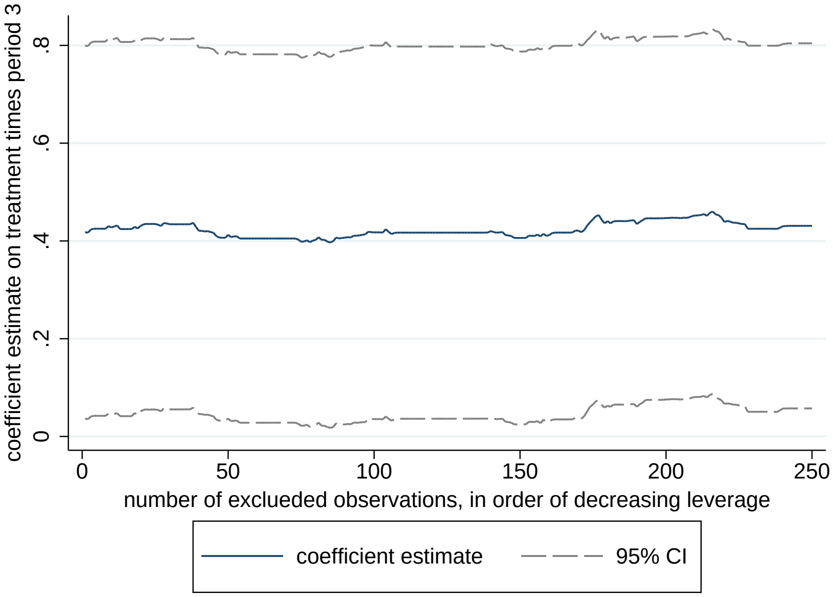

the far right point 4, 480 (= 4 × 120) of the shortest observations are omitted, or 8.6% of the sample. Results are presented in panel (a) with height-for-age as the dependent variable and in panel (b) with height in centimeters, for robustness. The

FIGURE B3. Effect estimate is robust to omitting the shortest children in each age×sex bin

(a) Dependent variable is height-for-age z-score

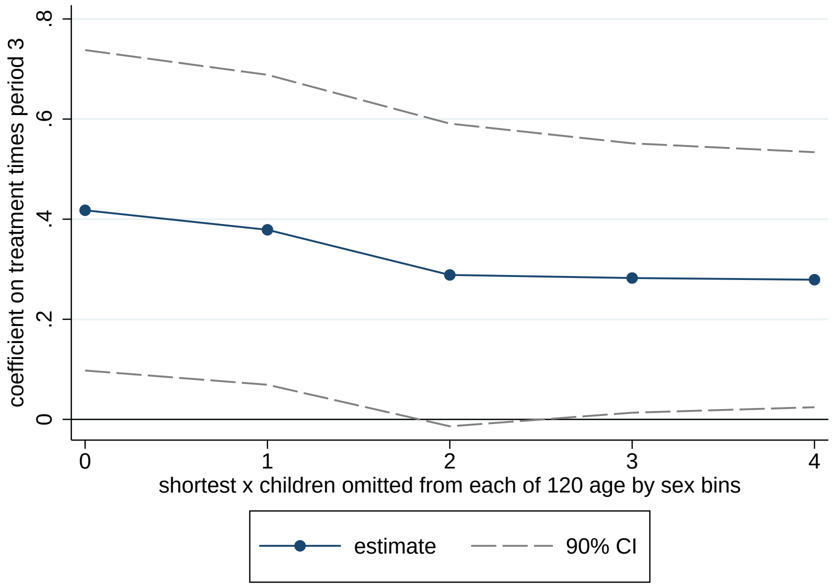

(b) Dependent variable is ln(height in centimeters)

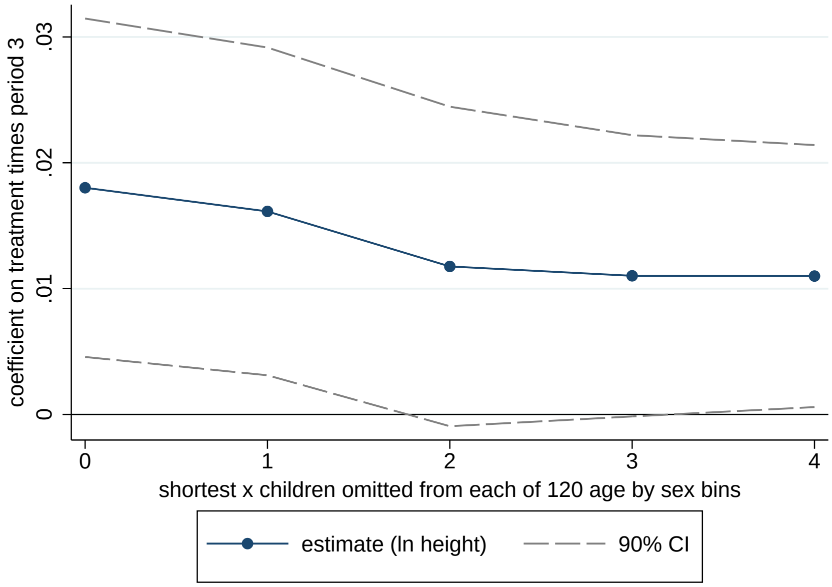

stability of the coefficient estimates suggests that our result is not driven by outlier data from a few extremely short (or apparently extremely short) children.

TABLE B2. Results are robust to omitting outliers within each age-in-months × sex bin

	(1)	(2)	(3)	(4)	(5)	(6)
dependent variable:	height-for-age	height-for-age	height-for-age	ln(height)	ln(height)	ln(height)
sample:	full	not small	not large	full	not small	not large
treatment	-0.0988	0.0186	-0.190	-0.00533	0.000230	-0.00874
	(0.129)	(0.129)	(0.131)	(0.00572)	(0.00550)	(0.00586)
treatment × period 2	0.236†	0.112	0.304*	0.00973	0.00415	0.0122†
	(0.140)	(0.124)	(0.151)	(0.00618)	(0.00528)	(0.00666)
treatment × period 3	0.418*	0.323†	0.359†	0.0180*	0.0132†	0.0158†
	(0.195)	(0.188)	(0.185)	(0.00817)	(0.00775)	(0.00796)
round FEs	✓	✓	✓	✓	✓	✓
age × sex FEs	✓	✓	✓	✓	✓	✓
n	3,432	3,320	3,365	3,432	3,320	3,365

B3.2.2. *Dropping observations more than 1.96 standard deviations from the mean of each age-sex bin.* Table B2 presents results from an alternative, less arbitrary, approach to excluding extreme values. Within each of the 120 age-in-months by sex bins, the mean and standard deviation of those observations was computed. Because there are 3,432 observations, the average bin has 28.6 observations. Exceptionally short observations are those more than 1.96 standard deviations below the mean of their bin (using the standard deviation of their bin); exceptionally tall observations are those more than 1.96 standard deviations above the mean of their bin.

Columns 2 and 3 of table B2 present results omitting the exceptionally small and exceptionally large observations, respectively. Columns 4 through 6 replicate these regressions using the log of height in centimeters as the dependent variable, instead of height-for-age z-scores. In all cases, the main result is qualitatively preserved, even on this restricted sample. This approach offers no evidence that our result is merely driven by exceptionally short children.

B4. Comparison with an alternative choice of truncation points

B4.1. **Evaluation against a normal distribution.** About 4 percent of our main sample has height-for-age z-scores between -8 and -6; as we have seen, this fraction is smaller than the corresponding fraction in the IHDS and larger than that in the DHS (2.4 percent below -6 and 1.4 percent between -8 and -6), when computed using the same Stata program. This section considers the consequences of using the alternative cutpoint of -6 to 6, matching the truncation enforced into the coded DHS, rather than our main analysis cutpoint of -8 to 4. For transparency, Supplementary Appendix A presented results for many combinations of cut-points.

One standard by which to judge the plausibility of height data is its normality. Before proceeding with this analysis, we note that a quantile-quantile (Q-Q) plot against normality fails to recommend the alternative cutpoints of -6 to 6. As figure S2 shows, our preferred cutpoints of -8 to 4 visibly more closely match a normal distribution than does the alternative considered in this section.

Figure B4. Q-Q plot against normality of our data using preferred and alternative cutpoints

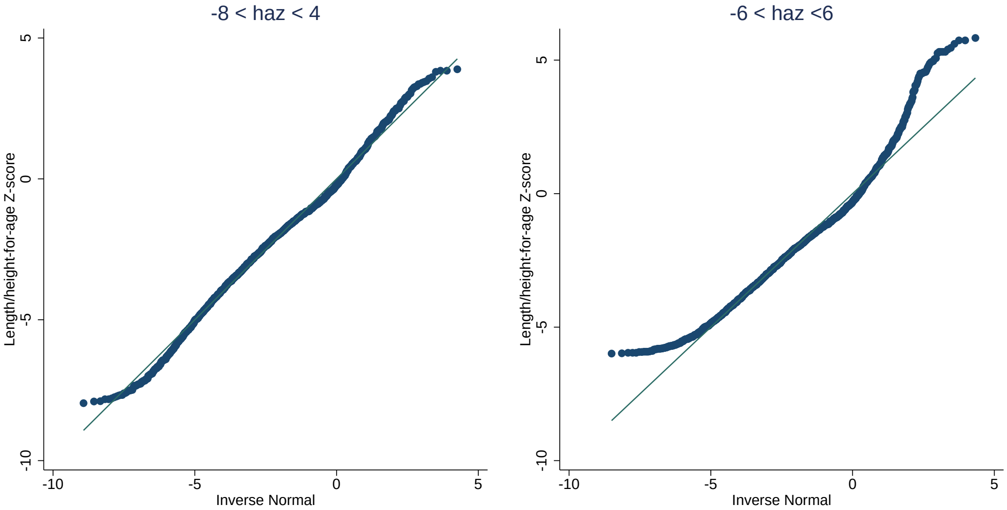

Table B3. Count of appearances of a child across rounds of our data is associated with dispersion in measured height

	number of rounds in which child appears		
	in 1 round	in 2 rounds	in 3 rounds
std. dev. of height-for-age	1.99	1.84	1.75
std. dev. of residuals after age and sex	2.09	1.86	1.81

B4.2. **Estimates of treatment effect with alternative truncation.** Despite the closer match of our preferred -8 to 4 truncation point to a normal distribution, we here consider whether and in which specifications use of the alternative -6 to 6 cutpoints produces similar results to use of our preferred cutpoints.

As discussed in the text, the experiment we study was designed as a panel of villages, not a panel of children. However, some children were tracked through all three rounds. Children who were young within the interval 0-5 at baseline and old within this interval at endline were most likely to be tracked, by construction of the age structure of the sample. Although our main results in the paper respect the original intent of the study designers that this experiment be studied as a panel of villages, for robustness we also show results in the paper exploiting the child-level panel structure of the subset of the sample that was tracked; in this panel analysis, the main text reports, we find similar results.

This supplement has used dispersion of the height-for-age measure as a measure of data quality. In table B3, we note that children who were observed in more rounds have higher-quality height data, as measured by the standard deviation of height-for-age. This is not merely because they are more age-homogenous, because this pattern persists in residuals after 120 dummies for age-in-months by sex, the level at which height-for-age scores are constructed. This may be because families

Table B4. Estimates of treatment effect with alternative truncation, various strategies

	(1)	(2)	(3)	(4)
truncation:	-8 to 4	-6 to 6	-6 to 6	-6 to 6
level:	child	child	child	village
panel structure:	none	none	if in > 1	within child, 1 to 3
n	3,432	3,339	2,305	$247 \times 2 = 494$
Panel A: Height-for-age z-scores as dependent variable				
treatment	-0.0988	0.116	0.00688	
	(0.129)	(0.147)	(0.179)	
treatment round 2	0.236	0.0980	0.215	
	(0.140)	(0.154)	(0.183)	
treatment round 3	0.418	0.181	0.375	0.473
	(0.195)	(0.197)	(0.210)	
effect p-value:	0.036	0.36	0.078	0.052
Panel B: Dichotimized stunting as dependent variable				
treatment	-0.0126	-0.0343	0.00811	
	(0.0348)	(0.0382)	(0.0443)	
treatment round 2	-0.00444	0.00928	-0.00715	
	(0.0393)	(0.0418)	(0.0482)	
treatment round 3	-0.0705	-0.0465	-0.122	-0.135
	(0.0487)	(0.0501)	(0.0607)	
effect p-value:	0.153	0.36	0.049	0.098

In column 4, within-child differences are collapsed into village average differences, and the p-values is computed from a non-parametric Komolgorov-Smirnov test on a sample of $n = 60$ villages.

with more experience with the survey team and measurement procedures were more cooperative with height measurements (for example, helping to hold children still on the measuring board) or because higher-quality surveyors both measured height more carefully and were more diligent in tracking down panel members. Whatever the explanation, this association provides a data-driven reason to consider the panel subset of the data in this analysis of data quality and dispersion – noting that some readers may in any event prefer the internal validity of the child-level panel.

Table B4 presents alternative estimates of the effect of the program using -6 to 6 truncation. Every regression is replicated with both height-for-age and dichotomized stunting as the dependent variable. Column 1 reprints the main estimate of the paper. Column 2 reprints the estimate from supplementary appendix section A4; column 5 conducts similar analysis for dichotomized stunting. Averaging over many sets of cutpoints, that table found an average treatment effect of 0.30 with an average t of 1.96. However, in this cases the estimate of the effect is smaller in absolute value and not statistically significantly different from zero.

Columns 3 and 4 report finding effects comparable to our main estimates using the alternative -6 to 6 cutpoints, in the higher quality (less dispersed) subsample where children were measured longitudinally in multiple panel rounds, both for

continuous z-scores and for dichotomized stunting. Columns 3 merely excludes observations that appear in only one round and repeating the main analysis.

Column 4 reports a conservative non-parametric analysis. First, for each panel it computes within-child differences in height-for-age from rounds 1 to 3. Next, it collapses these differences in the village means; this produces the average effect. Finally, it conducts a non-parametric test to verify the significance of the small-sample difference between the treatment and control groups in these average height differences. The regressions and the non-parametric approach both find results that are similar to one another and to our main results. Note that within-child differencing would be expected to produce more precise estimates of child fixed effects remove fixed heterogeneity in child-specific genetic potential height.

Therefore, although we believe it is correct to prefer the -8 to 4 sample, and having considered in Supplementary Appendix A the sensitivity and robustness of our conclusions to a large matrix of possible alternative cut-points, here we show that in the alternative -6 to 6 sample there is some evidence in support of our estimated treatment effect, although to be sure it depends on the specification. We particularly see a comparable effect size if the panel structure of the highest-quality data is fully exploited.

B5. Conclusion

As in many studies where the data were not collected by the researchers personally, it is not ultimately possible for us to verify conclusively that the height data that we use were collected correctly; we do not have any record of the interaction between a surveyor and a child that produced our data beyond the dataset iteslf. Moreover, it is clear that height as measured in our poor, rural sample is considerably more dispersed than in the WHO's healthy reference population. Some of this dispersion certainly reflects the variance introduced by negative health shocks; some of it may well be measurement error. In Supplementary Appendix A, we documented how our results sometimes are and sometimes are not influenced by a full set of 36 alternative truncation cutpoints for our height sample. Here, we compare our sample with other datasets and focus on the role of extremely short measured heights and of an alternative -6 to 6 threshold. This supplementary appendix has presented evidence that there is no reason to conclude that our main results merely reflect questionable height data or the influence of a few apparently extremely short observations.
